# Inhibiting S-palmitoylation arrests metastasis by relocating Rap2b from plasma membrane in colorectal cancer

**DOI:** 10.1038/s41419-024-07061-2

**Published:** 2024-09-14

**Authors:** Jiangli Zhu, Xize Cao, Zhenshuai Chen, Birou Lai, Lingling Xi, Jinghang Zhang, Shaohui Zhu, Shiqian Qi, Yinming Liang, Fei Cao, Binhui Zhou, Yu Song, Sheng Jiang, Tianyu Wang, Xiaohong Kang, Eryan Kong

**Affiliations:** 1 The First Affiliated Hospital of Xinxiang Medical University, Xinxiang, China; 2 Institute of Psychiatry and Neuroscience, Xinxiang Key Laboratory of Protein Palmitoylation and Major Human Diseases, Xinxiang Medical University, Xinxiang, China; 3 Lankao County Central Hospital, Lankao, China; 4 Henan Health Commission Key Laboratory of Gastrointestinal Cancer Prevention and Treatment, Xinxiang Medical University, Xinxiang, China; 5 State Key Laboratory of Biotherapy and Cancer Center, West China Hospital, Sichuan University and National Collaborative Innovation Center, Chengdu, China; 6 College of Pharmacy, Xinxiang Medical University, Xinxiang, China; 7 School of Pharmacy, China Pharmaceutical University, Nanjing, China

**Keywords:** Sumoylated proteins, Cancer epigenetics

## Abstract

Rap2b, a proto-oncogene upregulated in colorectal cancer (CRC), undergoes protein S-palmitoylation at specific C-terminus sites (C176/C177). These palmitoylation sites are crucial for Rap2b localization on the plasma membrane (PM), as mutation of C176 or C177 results in cytosolic relocation of Rap2b. Our study demonstrates that Rap2b influences cell migration and invasion in CRC cells, independent of proliferation, and this activity relies on its palmitoylation. We identify ABHD17a as the depalmitoylating enzyme for Rap2b, altering PM localization and inhibiting cell migration and invasion. EGFR/PI3K signaling regulates Rap2b palmitoylation, with PI3K phosphorylating ABHD17a to modulate its activity. These findings highlight the potential of targeting Rap2b palmitoylation as an intervention strategy. Blocking the C176/C177 sites using an interacting peptide attenuates Rap2b palmitoylation, disrupting PM localization, and suppressing CRC metastasis. This study offers insights into therapeutic approaches targeting Rap2b palmitoylation for the treatment of metastatic CRC, presenting opportunities to improve patient outcomes.

## Introduction

Rap2b, a member of the Ras superfamily [1–3], was initially discovered in human platelet [3–5]. It has since been identified as a proto-oncogene involved in promoting proliferation and invasion in various tumors [2, 6, 7]. Recent studies have demonstrated that Rap2b is highly upregulated in CRC [7, 8]. Importantly, when comparing between CRC cases with or without lymph node metastasis, Rap2b was found to be specifically elevated in the invaded lymph nodes [8], suggesting a crucial role for Rap2b in the regulation of metastasis in CRC. Clinically, metastatic CRC remains an incurable disease, with common sites of metastasis including the liver, lungs, or lymph nodes and peritoneum. The median overall survival for patients is approximately 12 months, with a 5-year survival rate of <20% [9, 10].

Akin to H-/N-Ras, Rap2b possesses three distinct segments: a protein interacting motif on the N-terminus, a GTP/GDP binding motif in the middle, and a C-terminus that features a CAAX motif on the end [11, 12]. Notably, the CAAX motif contains the capability to recruit versatile lipid modifications, such as palmitoylation, for membrane localization [12]. Indeed, studies have indicated that Rap2b is enriched in the brush border membrane of intestinal epithelial cells and undergoes cycling between the granule membrane and plasma membrane (PM) in neutrophils [13, 14]. However, the detailed regulatory mechanism governing its dynamic subcellular localization and function remains unknown.

Protein S-palmitoylation, also known as palmitoylation, is a reversible lipid modification that plays a crucial role in modulating various aspects of targeted proteins, particularly their membrane localization. This modification involves the attachment of palmitic acid to cysteine residues of the protein through thioester linkage, which enhances the hydrophobicity of the modified protein [15–17]. The dynamics of protein palmitoylation is achieved through a cycle of palmitoylation, catalyzed by palmitoyltransferase ZDHHCs 1–9 and 11–25 (with no known isoform 10), and depalmitoylation, catalyzed by depalmitoylase APT1/2, PPT1/2, and ABHD17a [18–21].

In an investigation of palmitoylated proteins in our laboratory, we discovered that Rap2b is likely modified through protein palmitoylation. This finding led us to speculate whether Rap2b undergoes palmitoylation in colorectal cancer (CRC) and, furthermore, if palmitoylation of Rap2b (referred to as palm-Rap2b) is involved in regulating its dynamic subcellular localization and function. Our study demonstrates that palmitoylation is vital for the PM localization of Rap2b and its role in cell migration and invasion. Importantly, when we blocked Rap2b palmitoylation using a high-binding-affinity peptide (PTG-101), we observed inhibition of CRC metastasis both in vitro and in vivo. These findings suggest that targeting palmitoylation could be a valuable therapeutic strategy for suppressing Rap2b signaling, and PTG-101 emerges as a potent drug candidate for intervening in metastatic CRC in clinical settings.

## Results

### Rap2b is associated with a poor prognosis in CRC and is palmitoylated at cysteine-176 and cysteine-177

**Fig. 1:**
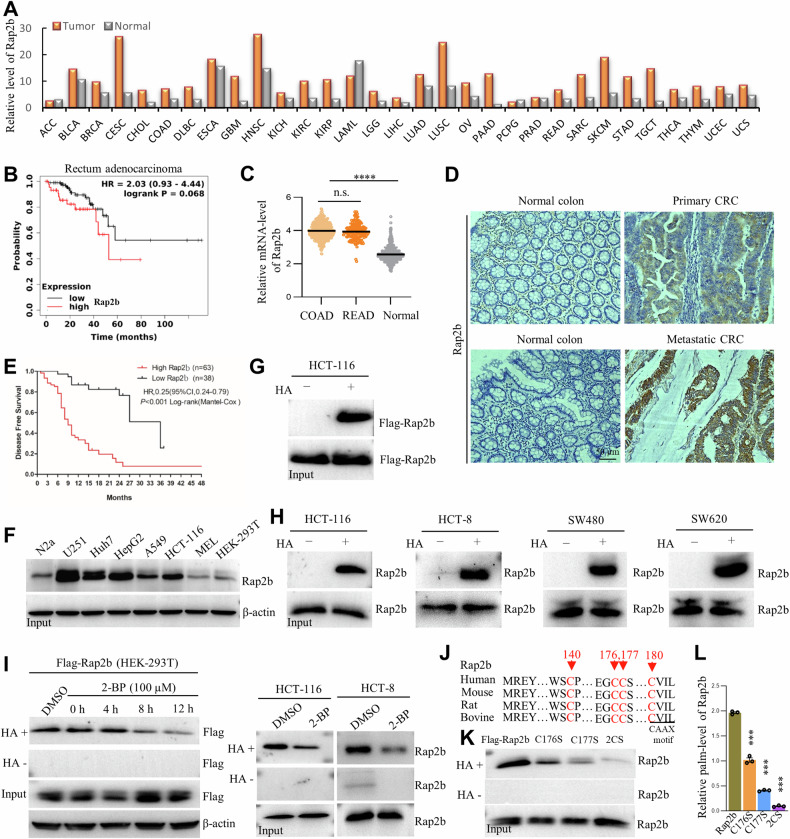
Rap2b is associated with a poor prognosis in CRC and is palmitoylated at cysteine-176 and cysteine-177. **A** Upregulation of Rap2b in various types of cancers (data from TCGA). **B** Negative correlation between high Rap2b expression and survival in rectum adenocarcinoma (TCGA). **C** Comparison of Rap2b mRNA levels between Colorectal cancer and normal tissues (TCGA). **D** Immunostaining of Rap2b in paraffin sections of colon cancer to evaluate protein expression. **E** Kaplan–Meier analysis of Rap2B expression and disease-free survival (DFS) in CRC patients. ***P < 0.001, *n* = 101. **F** Evaluation of endogenous Rap2b levels in various cancer cell lines by WB. **G**, **H** RAC assay to examine protein palmitoylation of ectopically expressed Flag-Rap2b or endogenously expressed Rap2b. HA− without hydroxylamine; HA+ with hydroxylamine. **I** RAC assay to examine protein palmitoylation of ectopically expressed Flag-Rap2b or endogenously expressed Rap2b treated with DMSO or 2-BP. **J** Protein sequence alignment of Rap2b for conservation analysis. **K**, **L** RAC assay and quantification of Rap2b and its mutants for protein palmitoylation. *n* = 3 biological replicates, one-way ANOVA followed by Tukey’s post hoc test, ****P < 0.0001. Data are presented as mean ± S.E.M.

Initially, the expression profile of Rap2b was examined using the datasets from The Cancer Genome Atlas Program (TCGA). The results indicated that Rap2b is generally upregulated in multiple types of tumors (Fig. 1A). Further analysis suggested that a higher level of Rap2b is negatively correlated with the survival of cancer patients, particularly in rectum carcinoma (Fig. 1B). Consistently, the relative mRNA level of Rap2b was significantly upregulated in colorectal carcinoma (Fig. 1C). Additionally, to evaluate the clinical significance of Rap2B, we analyzed the expression of Rap2B protein in 117 paired CRC tissues. Immunohistochemical (IHC) staining revealed that Rap2B was significantly increased in CRC compared to adjacent tissues (Fig. 1D). The expression of Rap2B positively correlated with higher UICC/AJCC TNM stages, lymph node metastasis, and distant metastasis (Table S1). Kaplan–Meier analysis demonstrated that high expression of Rap2B correlated with reduced disease-free survival (DFS) in CRC patients in an independent cohort (Fig. 1E). Univariate and multivariate Cox analysis showed that Rap2B expression independently correlated with DFS in CRC patients (Table S2). These data suggest that Rap2b might play a central role in CRC metastasis.

To verify whether Rap2b is palmitoylated, several cancer cell lines were used. The results showed that Rap2b is highly expressed in various cell lines, including HCT-116, a human CRC cell line (Fig. 1F and Supplementary Fig. 1). Next, ectopically or endogenously expressed Rap2b was examined using the Acyl-RAC assay (RAC) to evaluate protein palmitoylation. The results showed that Rap2b is readily palmitoylated both in vitro and in vivo (Fig. 1G, H and Supplementary Fig. 1). Moreover, 2-bromohexadecanoic acid (2-BP), an inhibitor of protein palmitoylation, was incubated with cells expressing Rap2b, and the results showed that 2-BP could steadily reduce the level of palm-Rap2b (Fig. 1I and Supplementary Fig. 1), indicating that Rap2b palmitoylation is reversible.

Since reversible palmitoylation occurs only on cysteine residues, the Rap2b protein sequence was aligned, and a total of four cysteines (C140/C176/C177/C180) were identified, all of which are evolutionarily conserved (Fig. 1J). To identify the specific site of palmitoylation, Flag-Rap2b was purified (Fig. S1A) and subjected to mass spectrometry (MS) analysis. The MS data demonstrated that the peptide containing C140 was captured multiple times (94), but no positive signal of palm-C140 was detected (Fig. S1B), indicating that C140 is probably not palmitoylated. Unfortunately, the remaining cysteine residues are located at the very end of the C-terminus, which were not captured in the MS analysis (Fig. S1C). Alternatively, the strategy of point mutation was applied, and the results showed that both C176 and C177 are modified with palmitoylation: as both mutations (C176S or C177S) significantly lower the level of palm-Rap2b, and the double mutant (C176S/C177S, 2CS) almost completely abolishes palmitoylation (Fig. 1K, L and Supplementary Fig. 1). Consistently, the mPEG labeling assay confirmed that Rap2b displayed two distinct migrating bands in the +HA lane (caused by the substitution of 5KD mPEG for palmitate) (Fig. S1D).

Together, the above data demonstrate that CRC progression is tightly correlated with the high expression of Rap2b, and the latter is specifically modified at C176 and C177 for palmitoylation.

### Palmitoylation is required for the membrane localization of Rap2b

**Fig. 2:**
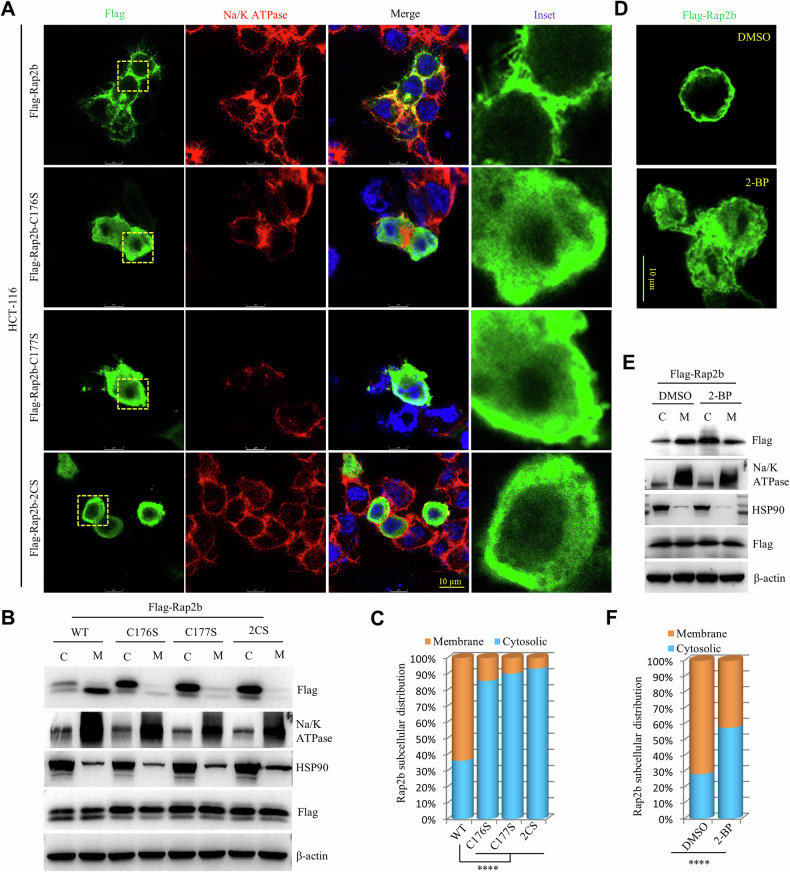
Palmitoylation is required for the membrane localization of Rap2b. **A** Immunofluorescence microscopy of HCT-116 cells expressing Rap2b or its mutants. Na/K-ATPase was used as a marker for the plasma membrane. **B**, **C** Cytosol/membrane fractionation and western blot analysis of HCT-116 cells expressing Rap2b or its mutants. Quantification of the results is shown. Statistical analysis was performed using one-way ANOVA followed by Tukey’s post hoc test. ****P < 0.0001. HSP90 was used as a marker for the cytosol. **D** Immunofluorescence microscopy of HCT-116 cells expressing Flag-Rap2b treated with either DMSO or 2-BP. **E**, **F** Cytosol/membrane fractionation and western blot analysis of HCT-116 cells expressing Flag-Rap2b treated with either DMSO or 2-BP. Quantification of the results is shown. Statistical analysis was performed using a two-tailed *t*-test. ****P < 0.0001. Data are presented as mean ± S.E.M.

To determine whether palmitoylation regulates the membrane localization of Rap2b, we expressed wild-type (WT) Rap2b and the Rap2b-2CS mutant in HCT-116 cells. Immunofluorescence images revealed that Rap2b is primarily associated with the PM. However, when palmitoylation was blocked by introducing the C176S, C177S, or 2CS mutations, the PM localization of Rap2b was completely abolished, and the mutant proteins were relocated to the cytosol (Fig. 2A). To confirm these findings, we used a biochemical approach to isolate the cytosol and membrane fractions from these cells. Consistently, the results demonstrated that while WT-Rap2b predominantly localizes to the membrane fraction (M), the mutant proteins (C176S, C177S, or 2CS) showed a shift in localization from the membrane to the cytosol (C) (Fig. 2B, C and Supplementary Fig. 1).

To confirm that the observed shift in subcellular localization of Rap2b is indeed due to the inhibition of palmitoylation and not a result of the cysteine-to-serine mutations, we treated HCT-116 cells expressing Rap2b with 2-BP, a known inhibitor of palmitoylation. Consistently, the experiment demonstrated that treatment with 2-BP mimicked the effects of the Rap2b-2CS mutation, leading to a shift in the subcellular localization of Rap2b from the PM to the cytosol (Fig. 2D–F and Supplementary Fig. 1). Furthermore, similar results were obtained in HCT-8 cells, reinforcing the conclusion that the palmitoylation of C176/C177 is necessary for the PM localization of Rap2b in CRC cells (Fig. S2A, B).

### Role of Rap2b palmitoylation in migration and invasion of CRC cells

**Fig. 3:**
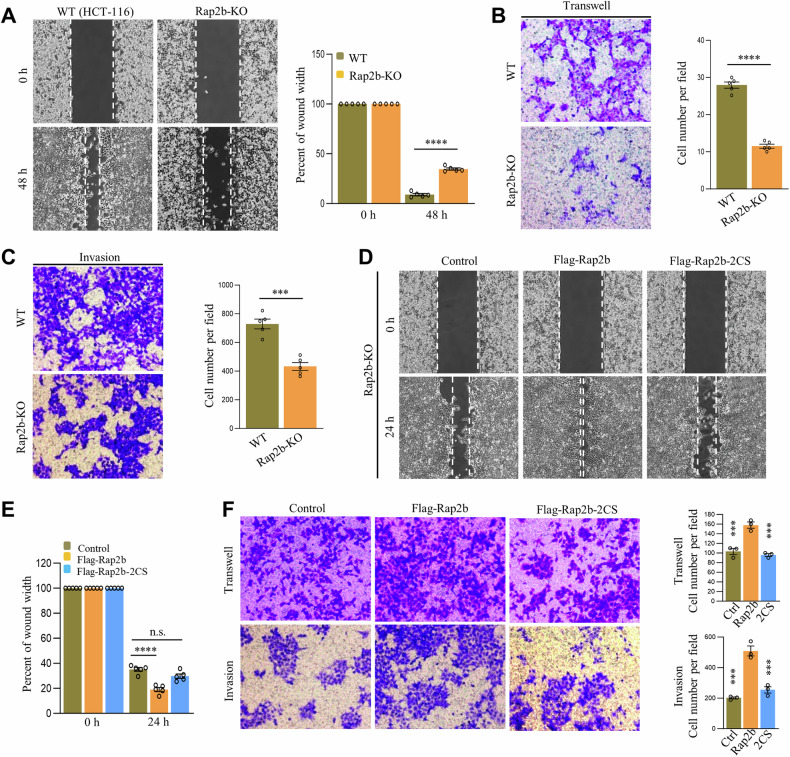
Role of Rap2b palmitoylation in migration and invasion of CRC cells. **A** Wound-healing analysis of wild-type (WT) and Rap2b knockout (Rap2b-KO) HCT-116 cells, with quantification. Statistical analysis was performed using a two-tailed *t*-test. ****P < 0.0001. *n* = 5 biological replicates. **B** Transwell analysis of WT and Rap2b-KO HCT-116 cells, with quantification. Statistical analysis was performed using a two-tailed *t*-test. ****P < 0.0001. *n* = 5 biological replicates. **C** Matrigel invasion analysis of WT and Rap2b-KO HCT-116 cells, with quantification. Statistical analysis was performed using a two-tailed *t*-test. ***P < 0.001. *n* = 5 biological replicates. **D**, **E** Wound-healing assay of Rap2b-KO cells rescued with either Rap2b or Rap2b-2CS, with quantification (**E**). Statistical analysis was performed using one-way ANOVA followed by Tukey’s post hoc test. ****P < 0.0001. *n* = 5 biological replicates. **F** Matrigel invasion assay of Rap2b-KO cells rescued with either Rap2b or Rap2b-2CS, with quantification. Statistical analysis was performed using one-way ANOVA followed by Tukey’s post hoc test. ***P < 0.001. *n* = 3 biological replicates. Data are presented as mean ± S.E.M.

To investigate the involvement of Rap2b and its palmitoylation in CRC, we generated a Rap2b knockout (KO) cell line in HCT-116 cells (Fig. S3A–E). Several approaches, including wound-healing, transwell, and matri-gel invasion assays, were employed to evaluate the effects. Our findings revealed that the deletion of Rap2b significantly impairs cell migration and invasion compared to the control (Fig. 3A–C). However, Rap2b-KO cells did not exhibit significant alterations in proliferation compared to the control (Fig. S4A). Similarly, ectopic expression of Rap2b did not appear to affect cell proliferation in HCT-116 cells (Fig. S4B).

To specifically assess the role of Rap2b palmitoylation, we expressed Flag-Rap2b and Flag-Rap2b-2CS (a non-palmitoylated mutant) in Rap2b-KO cells for rescue experiments. The wound-healing, transwell, and matri-gel assays demonstrated that the expression of Rap2b, but not Rap2b-2CS, promoted cell migration and invasion (Fig. 3D–F). Similarly, the expression of Flag-Rap2b and Flag-Rap2b-2CS in WT HCT-8 cells yielded similar results, indicating that the enhancement of cell migration and invasion is dependent on the palmitoylation of Rap2b (Fig. S4C–F).

Collectively, these results indicate that palmitoylation is necessary for Rap2b to regulate cell migration and invasion, but not proliferation, in CRC cells.

### ABHD17a-mediated depalmitoylation relocates Rap2b from the plasma membrane and regulates cell migration and invasion

**Fig. 4:**
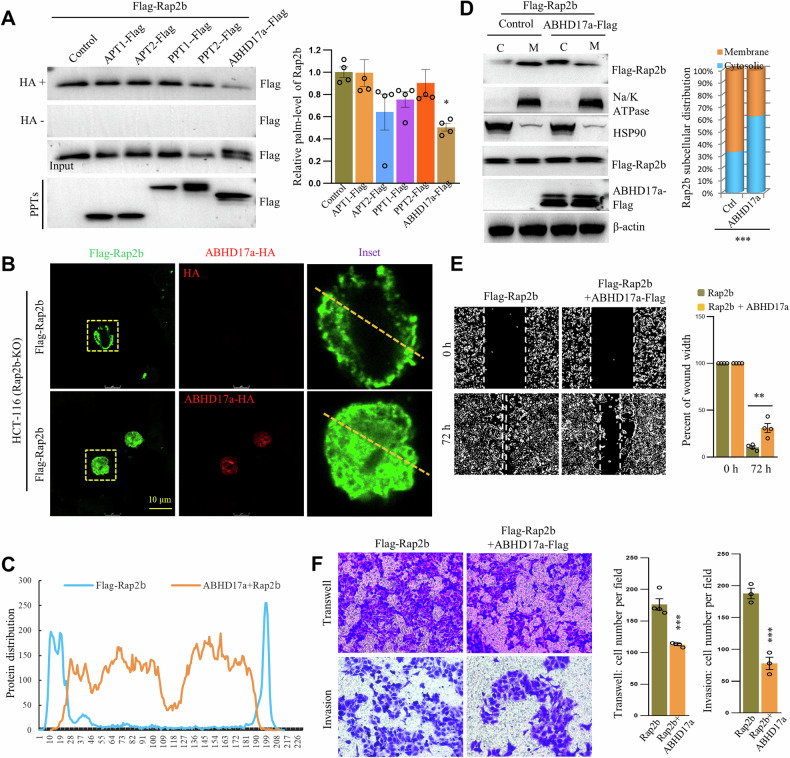
ABHD17a-mediated depalmitoylation relocates Rap2b from the plasma membrane and regulates cell migration and invasion. **A** Various thioesterases were coexpressed with Rap2b in HCT-116 cells, and the level of palmitoylated Rap2b (palm-Rap2b) was analyzed by RAC assay and quantified. Statistical analysis was performed using one-way ANOVA followed by Tukey’s post hoc test. *P < 0.05. *n* = 3 biological replicates. Fluorescence microscopy of HCT-116 cells (Rap2b-KO) coexpressing Flag-Rap2b with or without ABHD17a (**B**), with the distribution of Rap2b profiled (**C**). **D** Cytosol/membrane fractionation of HCT-116 cells (Rap2b-KO) coexpressing Flag-Rap2b with or without ABHD17a, analyzed by western blot and quantified. Statistical analysis was performed using a two-tailed *t*-test. ***P < 0.001. *n* = 3 biological replicates. Wound-healing (**E**), Transwell, and Matrigel invasion analysis (**F**) of HCT-116 cells (Rap2b-KO) coexpressing Flag-Rap2b with or without ABHD17a, with quantification. Statistical analysis was performed using a two-tailed *t*-test. **P < 0.01, ***P < 0.001. *n* = 3 biological replicates. Data are presented as mean ± S.E.M.

To elucidate the mechanism of Rap2b palmitoylation dynamics, we aimed to identify the enzyme responsible for depalmitoylating Rap2b. We coexpressed individual thioesterases (APT1/2, PPT1/2, and ABHD17a) with Rap2b and evaluated the levels of palm-Rap2b. The results revealed that only the coexpression of ABHD17a significantly reduced the levels of palm-Rap2b compared to the control (Fig. 4A and Supplementary Fig. 1). Since palmitoylation is required for the PM localization of Rap2b, we hypothesized that ABHD17a might modulate the PM localization of Rap2b. Indeed, immunofluorescence images showed that the coexpression of ABHD17a and Rap2b in both Rap2b-KO (HCT-116) and WT HCT-8 cells resulted in the relocation of Rap2b from the PM to the cytosol (Fig. 4B, C and Fig. S5A). Biochemically, while Rap2b was primarily located in the membrane fraction, the coexpression of ABHD17a caused the shift of Rap2b localization from the membrane to the cytosol (Fig. 4D, Fig. S5B, and Supplementary Fig. 1).

Functionally, it is logical to expect that the downregulation of palm-Rap2b would suppress cell migration and invasion. Indeed, wound-healing, transwell, and matri-gel assays demonstrated that the coexpression of ABHD17a with Rap2b hindered cell migration and invasion compared to the control (Fig. 4E, F).

In conclusion, our findings indicate that ABHD17a-mediated depalmitoylation relocates Rap2b from the PM to the cytosol, thereby attenuating cell migration and invasion in CRC cells.

### Palm-Rap2b regulates cell migration and invasion via EGFR/PI3K/AKT signaling

**Fig. 5:**
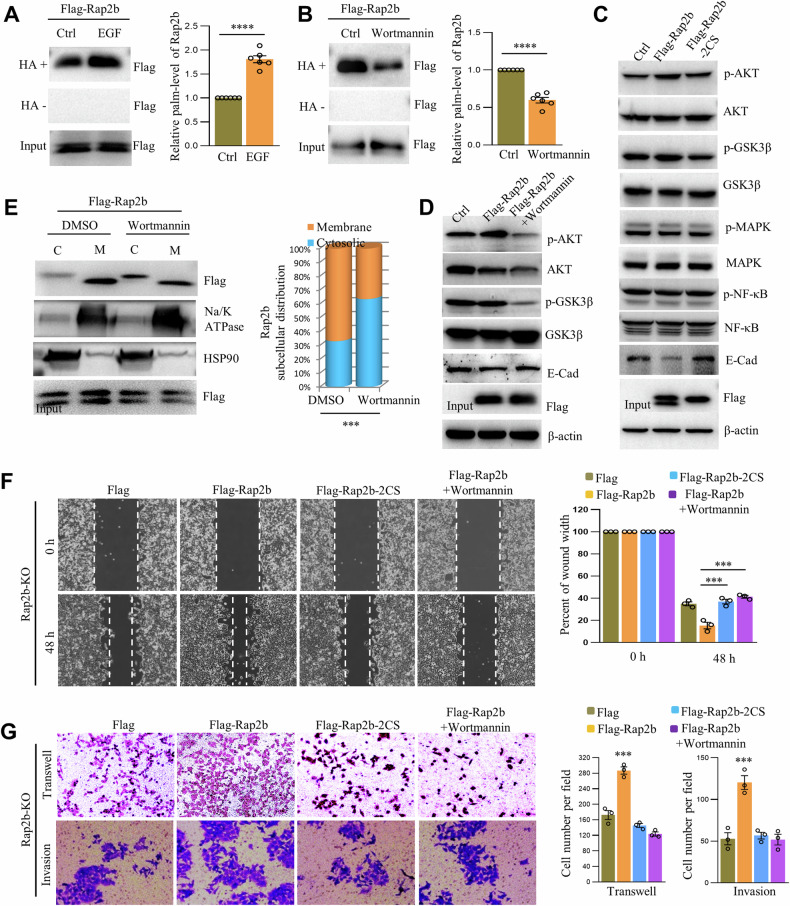
Palm-Rap2b regulates cell migration and invasion via EGFR/PI3K/AKT signaling. **A** HCT-116 cells (Rap2b-KO) expressing Flag-Rap2b were incubated with or without EGF for 12 h, and the level of palmitoylated Rap2b (palm-Rap2b) was analyzed by RAC assay and quantified. Statistical analysis was performed using a two-tailed *t*-test. ****P < 0.0001. *n* = 6 biological replicates. **B** HCT-116 cells (Rap2b-KO) expressing Flag-Rap2b were incubated with or without Wortmannin and analyzed by RAC assay for the level of palm-Rap2b, and quantified. Statistical analysis was performed using a two-tailed *t*-test. ****P < 0.0001. *n* = 6 biological replicates. **C** Cytosol/membrane fractionation of HCT-116 cells (Rap2b-KO) expressing Flag-Rap2b and treated with DMSO or Wortmannin, analyzed by western blot and quantified. Statistical analysis was performed using a two-tailed *t*-test. ***P < 0.001. *n* = 3 biological replicates. **D** Western blot analysis of HCT-116 cells (Rap2b-KO) expressing Flag-Rap2b or Flag-Rap2b-2CS. **E** Western blot analysis of HCT-116 cells (Rap2b-KO) expressing Flag-Rap2b and treated with DMSO or Wortmannin. **F** Wound-healing assay of HCT-116 cells (Rap2b-KO) expressing Flag-Rap2b or Flag-Rap2b-2CS and treated with DMSO or Wortmannin. Statistical analysis was performed using one-way ANOVA followed by Tukey’s post hoc test. ***P < 0.001. *n* = 3 biological replicates. **G** Transwell and matrigel invasion analysis of HCT-116 cells (Rap2b-KO) expressing Flag-Rap2b or Flag-Rap2b-2CS and treated with DMSO or Wortmannin. Statistical analysis was performed using one-way ANOVA followed by Tukey’s post hoc test. ***P < 0.001. *n* = 3 biological replicates. Data are presented as mean ± S.E.M.

Given that oncogenesis related to Ras family proteins is often mediated by EGFR signaling [22, 23], we investigated whether Rap2b palmitoylation is modulated by EGF. Interestingly, EGF significantly increased the levels of palm-Rap2b (Fig. 5A and Supplementary Fig. 1), while the incubation of Wortmannin (a PI3K inhibitor) dramatically suppressed palm-Rap2b (Fig. 5B and Supplementary Fig. 1), suggesting the involvement of the EGFR–PI3K axis. To confirm this, we examined the canonical EGFR signaling cascades (EGFR/MAPK or EGFR/PI3K/AKT) and found that the expression of Rap2b, but not Rap2b-2CS, activated p-AKT/p-GSK3β and downregulated E-Cadherin, but had no effect on MAPK/NF-kB signaling in HCT-116 cells (Fig. 5C and Supplementary Fig. 1). Similarly, the incubation of Wortmannin downregulated palm-Rap2b and deactivated p-AKT/p-GSK3β (Fig. 5D and Supplementary Fig. 1). Moreover, Wortmannin altered the subcellular distribution of Rap2b, relocating it from the membrane to the cytosol (Fig. 5E and Supplementary Fig. 1). Functionally, the incubation of Wortmannin with HCT-116 cells phenocopied the expression of Rap2b-2CS. Wound-healing, transwell, and matri-gel assays showed that both Wortmannin and Rap2b-2CS significantly inhibited cell migration and invasion compared to the control in Rap2b-KO cells rescued with WT-Rap2b (Fig. 5F, G).

Overall, these data suggest that the EGFR signaling cascade is involved in regulating Rap2b palmitoylation and modulates cell migration and invasion in HCT-116 cells.

### PI3K phosphorylates ABHD17a at S302 and Y229 to regulate Rap2b palmitoylation

**Fig. 6:**
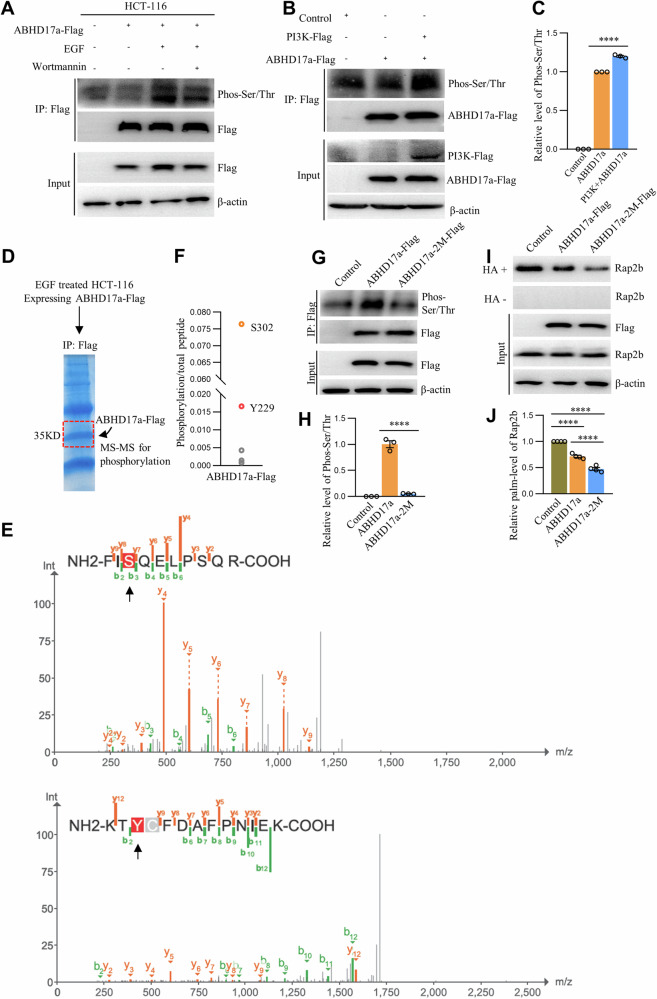
PI3K phosphorylates ABHD17a to regulate Rap2b palmitoylation. **A** HCT-116 cells expressing ABHD17a-Flag were treated with EGF alone or in combination with Wortmannin, and immunoprecipitated with Flag-agarose beads for western blot analysis. HCT-116 cells expressing Flag-ABHD17a alone or in combination with PI3K-Flag were immunoprecipitated with Flag-agarose beads for western blot analysis (**B**), and quantified (**C**). Statistical analysis was performed using one-way ANOVA followed by Tukey’s post hoc test. ****P < 0.0001. *n* = 3 biological replicates. Purified ABHD17a-Flag (**D**) was subjected to MS analysis to identify site-specific phosphorylation signals (**E**). **F** MS spectrum showing phosphorylation modifications at S302 and Y229. HCT-116 cells expressing ABHD17a-Flag or ABHD17a-2M-Flag were immunoprecipitated with Flag-agarose beads for western blot analysis (**G**), and quantified (**H**). Statistical analysis was performed using one-way ANOVA followed by Tukey’s post hoc test. ****P < 0.0001. *n* = 3 biological replicates. HCT-116 cells expressing ABHD17a-Flag or ABHD17a-2M-Flag were subjected to ABE analysis (**I**), and the level of Rap2b palmitoylation was quantified (**J**). Statistical analysis was performed using one-way ANOVA followed by Tukey’s post hoc test. ****P < 0.0001. *n* = 4 biological replicates. Data are presented as mean ± S.E.M.

Considering that palmitoylated Rap2b is localized on the PM, it is reasonable to speculate that ABHD17a may catalyze Rap2b depalmitoylation at the PM. This raises the question of how ABHD17a is activated at this site and whether it is related to EGFR/PI3K signaling. Therefore, we hypothesized that EGFR activates PI3K, which in turn phosphorylates ABHD17a, modulating its enzymatic activity and regulating Rap2b palmitoylation. To validate this hypothesis, ABHD17a-Flag was expressed in HCT-116 cells treated with EGF or Wortmannin. Immunoprecipitation of ABHD17a-Flag with Flag-agarose beads followed by analysis of pan Ser/Thr phosphorylation revealed that EGF enhances ABHD17a phosphorylation, while the combination of EGF and Wortmannin reduces ABHD17a phosphorylation (Fig. 6A and Supplementary Fig. 1), suggesting the involvement of the EGFR–PI3K axis in ABHD17a phosphorylation. Moreover, coexpression of PI3K significantly increased the level of ABHD17a phosphorylation (Fig. 6B, C and Supplementary Fig. 1).

To identify the specific phosphorylation site(s) in ABHD17a, purified ABHD17a-Flag (with EGF treatment) was subjected to MS analysis. The data indicated that S302 and Y229 are the top two candidate sites for phosphorylation (Fig. 6D–F). Subsequently, mutagenesis was performed, and the results demonstrated that ABHD17a-2M (S302A and Y229A) exhibited significantly reduced phosphorylation compared to WT ABHD17a (Fig. 6G, H and Supplementary Fig. 1), confirming that S302 and Y229 are the main phosphorylation sites in ABHD17a induced by EGF. Furthermore, to investigate how phosphorylation affects the enzymatic activity of ABHD17a, we found that ABHD17a-2M displayed higher enzymatic activity in reducing Rap2b palmitoylation compared to WT ABHD17a (Fig. 6I, J and Supplementary Fig. 1), suggesting that dephosphorylated ABHD17a exhibits increased enzymatic activity for depalmitoylation.

In conclusion, these results establish a connection between EGFR–PI3K signaling, ABHD17a phosphorylation, and Rap2b palmitoylation, highlighting the regulatory role of protein phosphorylation in the modulation of protein lipidation.

### Targeting palmitoylation inhibits migration and invasion by relocating Rap2b from plasma membrane

**Fig. 7:**
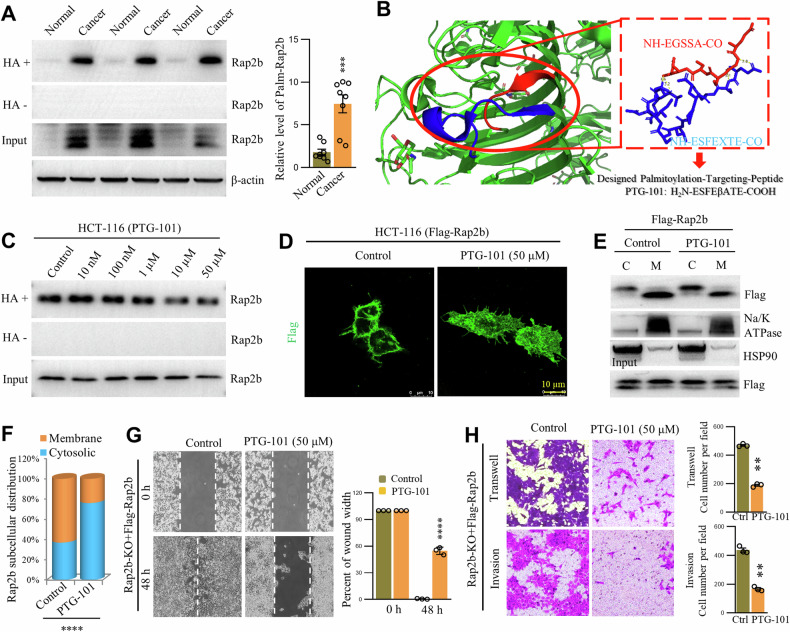
Targeting palmitoylation inhibits migration and invasion by relocating Rap2b from plasma membrane. **A** Colorectal cancer/normal tissue samples were collected for western blot analysis, and the level of Rap2b was quantified. Statistical analysis was performed using a two-tailed *t*-test. ****P < 0.0001. *n* = 3 biological replicates. **B** Design strategy of the peptide PTG-101, which binds to the palmitoylation peptide of Rap2b. PDB code: 1X0C. **C** HCT-116 cells were incubated with different concentrations of PTG-101, and the level of palmitoylated Rap2b (palm-Rap2b) was evaluated by RAC assay. **D** HCT-116 cells expressing Flag-Rap2b were treated with or without PTG-101 and analyzed by immunofluorescence microscopy. **E**, **F** HCT-116 cells expressing Flag-Rap2b were treated with or without PTG-101 and subjected to cytosol/membrane fractionation. The fractions were analyzed by western blot and quantified. Statistical analysis was performed using a two-tailed *t*-test. ****P < 0.0001. *n* = 3 biological replicates. **G** Rap2b-KO cells expressing Flag-Rap2b were treated with or without PTG-101 and subjected to a wound-healing assay. Statistical analysis was performed using a two-tailed *t*-test. ****P < 0.0001. *n* = 3 biological replicates. **H** Rap2b-KO cells expressing Flag-Rap2b were treated with or without PTG-101 and subjected to transwell and matrigel invasion assays. Statistical analysis was performed using a two-tailed *t*-test. **P < 0.01. *n* = 3 biological replicates. Data are presented as mean ± S.E.M.

To assess the potential therapeutic value of inhibiting palm-Rap2b in CRC, we examined the levels of Rap2b and palm-Rap2b in CRC tissue samples. Our results revealed elevated levels of both Rap2b and palm-Rap2b in CRC tissues compared to adjacent para-cancerous tissues (Fig. 7A and Supplementary Fig. 1). Based on this observation, we hypothesized that inhibiting palm-Rap2b could have beneficial effects in CRC. We employed two strategies to target the palmitoylation sites (C176/C177) of Rap2b. First, we designed peptides with high-binding affinity to the C-terminus of Rap2b (Fig. 7B and Fig. S6A, B). Second, we introduced a competitive peptide with the same protein sequence flanking C176/C177 (NH-EGCCSAC-CO, aa174-180). The results showed that the competitive peptide was not effective in attenuating the level of palm-Rap2b (Fig. S7A). In contrast, the designed peptide (H_2_N–ESFEβATE-COOH, named Palmitoylation-TarGeting-peptide 101, PTG-101) successfully suppressed Rap2b palmitoylation in a dose-dependent manner in HCT-116 and HCT-8 cells (Fig. 7C, Fig. S7A–C, and Supplementary Fig. 1). Interestingly, we compared the efficacy of PTG-101 with that of ABHD17a overexpression in reducing palm-Rap2b levels and found that PTG-101 at a dosage above 50 μM yielded similar results to ABHD17a ectopic expression in HCT-116 cells (Fig. S7B). At the cellular level, treatment with PTG-101 resulted in the relocation of Rap2b from the PM to the cytosol (Fig. 7D–F, Fig. S7E, F, and Supplementary Fig. 1). Importantly, PTG-101 significantly inhibited the migration and invasion of both HCT-116 and HCT-8 cells compared to the control (Fig. 7G, H and Fig. S7G, H). These findings highlight the potential of blocking palmitoylation as an effective strategy to suppress Rap2b function and suggest that PTG-101 is a potent peptide for inhibiting CRC metastasis.

Additionally, to test the applicability of extending these findings to other CRC cell lines such as SW480 and SW620 (two cell lines derived from the same patient but exhibiting markedly different invasive properties), experiments were conducted. Notably, these experiments revealed that both the expression level and palmitoylation of Rap2b are higher in SW620 compared to SW480 (Fig. S8A, B). Consistently, treatment with PTG-101 significantly downregulates palm-Rap2b levels in both SW480 and SW620 cells (Fig. S8C–F). Consequently, reduced palm-Rap2b levels, induced either by the Rap2b-2CS mutation or PTG-101 treatment, lead to the translocation of Rap2b from the PM to the cytosol (Fig. S8G–I). Functionally, PTG-101 treatment markedly suppresses the migration and invasion of SW480 and SW620 cells compared to the control condition (Fig. S8J–M). Taken together, these data suggest that the levels and intracellular localization of Rap2b in SW480 and SW620 are consistent with the conclusions drawn from HCT-116 cells.

According to the CMS1–4 classification [24], HCT-116 cells are classified as representative of CMS1 [25]. Notably, analysis of TCGA colon cancer data revealed differential expression of Rap2b across CMS1 to CMS4, with Rap2b showing the highest expression in CMS1 compared to the other subtypes (Fig. S9A). In normal colon cells, Rap2b exhibits weak expression from the bottom of the glands to the surface epithelium (Fig. S9B). Interestingly, regarding the impact of Rap2b levels on patient survival, the analysis indicated that a high level of Rap2b is negatively correlated with the survival of cancer patients in the CMS1–3 categories (*p* = 0.02 in CMS1), but not in CMS4 (Fig. S9C). Moreover, considering that metastasis is a multi-step process starting with the release of invasive cells from the primary tumor, we investigated whether migrating cells express more Rap2b than cells from the primary tumor itself. Our experiments indeed demonstrated that Rap2b expression is significantly higher in migrating cells compared to cells originating from the primary tumor (Fig. S9D, E).

### Inhibiting Rap2b palmitoylation by PTG-101 suppresses metastasis in xenograft mouse model of CRC

**Fig. 8:**
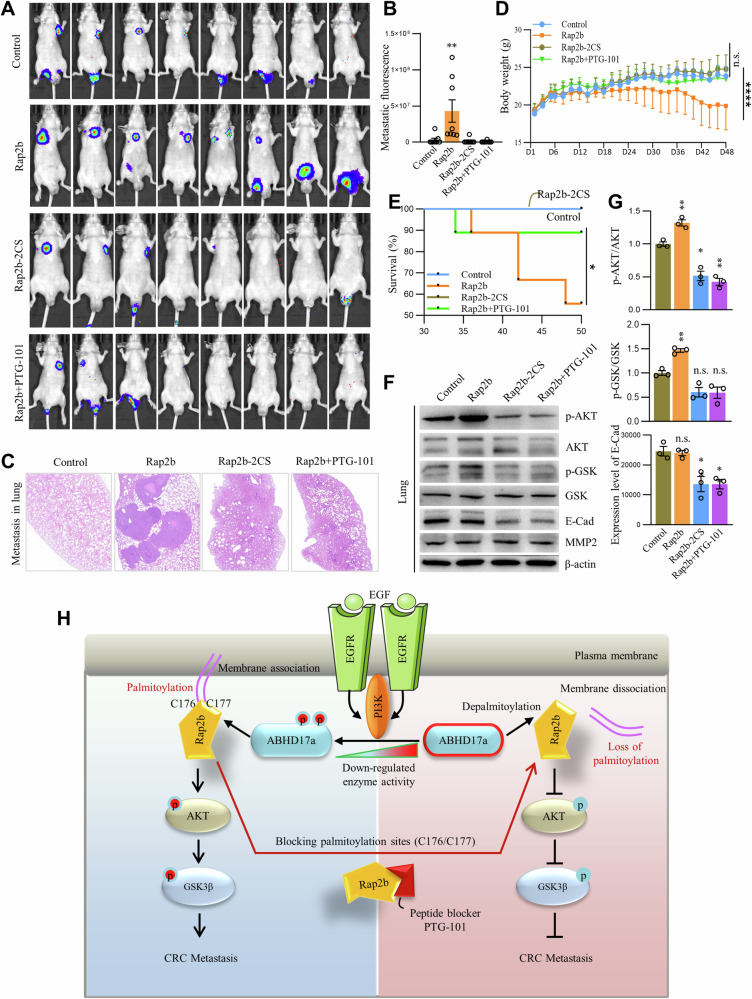
Inhibiting Rap2b palmitoylation by PTG-101 suppresses metastasis in xenograft mouse model of CRC. **A**, **B** HCT-116 cells expressing Rap2b or Rap2b-2CS were injected into the tail vein to establish a xenograft mouse model. One group was treated with PTG-101 and imaged for luciferase fluorescence at day 45. **C** Mouse lungs from different groups were paraffin-sectioned and subjected to HE staining. **D** Mouse body weight was recorded every other day throughout the establishment of the xenograft mouse model. Statistical analysis was performed using one-way ANOVA followed by Tukey’s post hoc test. ****P < 0.0001. *n* = 8 for each group. **E** Kaplan–Meier plot comparing lifespans between groups. *P* values were calculated using the log-rank test. *P < 0.05. *n* = 8 for each group. **F**, **G** Mouse lung homogenates from different groups were analyzed by western blot and quantified. Statistical analysis was performed using one-way ANOVA followed by Tukey’s post hoc test. **P < 0.01. *n* = 3 biological replicates. Data are presented as mean ± S.E.M. **H** Schematic representation of the potential pathological mechanism mediated by Rap2b palmitoylation to promote CRC metastasis. EGF/EGFR activates PI3K, leading to the phosphorylation of ABHD17a. This deactivates ABHD17a, resulting in elevated levels of palmitoylated Rap2b. Palmitoylated Rap2b enhances downstream signaling of AKT and GSK3β, promoting CRC metastasis. PTG-101, a peptide blocker of the palmitoylation sites (C176/C177) of Rap2b, inhibits Rap2b palmitoylation, causing Rap2b to be relocated from the plasma membrane. This disrupts signaling transmission, suppresses AKT and GSK3β phosphorylation, and ultimately inhibits CRC metastasis.

To investigate the potential of inhibiting Rap2b palmitoylation in suppressing CRC metastasis in vivo, we established xenograft mouse models by injecting Rap2b-KO cells expressing either GFP-Rap2b or GFP-Rap2b-2CS via the tail vein. We also administered PTG-101, packed with liposomes, via intraperitoneal injection at a daily dosage of 0.16 mg/20 g (resulting in a drug-in-blood concentration of ~80 µM). Our results revealed that the expression of Rap2b significantly enhanced CRC metastasis in the xenograft mouse model. However, the expression of Rap2b-2CS or treatment with PTG-101 effectively abolished this effect (Fig. 8A, B), as confirmed by histological analysis (Fig. 8C). Additionally, we observed a significant decrease in body weight and survival rate in the Rap2b group, but not in the Rap2b-2CS or PTG-101 treatment group, compared to the control group (Fig. 8D, E). Consistently, at the molecular level, the levels of p-AKT and p-GSK3β were significantly upregulated, while the level of E-Cadherin was downregulated in the Rap2b group, but not in the Rap2b-2CS or PTG-101 treatment group, compared to the control group (Fig. 8F, G and Supplementary Fig. 1).

Collectively, these findings demonstrate that Rap2b palmitoylation promotes CRC metastasis through modulation of the AKT/GSK3β signaling pathway, and inhibiting palm-Rap2b using PTG-101 effectively suppresses CRC metastasis in vivo.

## Discussion

The identification of Rap2b as a palmitoylated protein has led us to uncover a mechanism in which Rap2b palmitoylation facilitates cell migration and invasion in CRC cells, while not affecting cell proliferation. Mechanistically, we have demonstrated that Rap2b palmitoylation at C176 and C177 is crucial for its PM localization, which is regulated by upstream EGFR/PI3K signaling (Figs. 1 and 2). Additionally, we have shown that the depalmitoylase ABHD17a is responsible for the removal of Rap2b palmitoylation (Fig. 4), and that PI3K may phosphorylate and deactivate ABHD17a, leading to an increase in the level of palm-Rap2b (Figs. 5 and 6) and promotion of CRC metastasis through activation of the AKT-GSK3β signaling pathway (Fig. 3). Importantly, our strategy of inhibiting Rap2b palmitoylation using PTG-101, a peptide designed to block C176/C177 palmitoylation, successfully relocates Rap2b from the PM to the cytosol, inhibits AKT-GSK3β signaling, and suppresses CRC metastasis both in vitro and in vivo (Figs. 7 and 8). These findings highlight the significance of Rap2b palmitoylation in CRC and suggest that targeting palmitoylation holds therapeutic potential for intervening in metastatic CRC (Fig. 8H).

In addition, we have depicted the canonical signaling pathways downstream of EGFR, including PI3K/AKT/GSK3β and RAS/BRAF/MAPK. Targeting different components within these pathways often results in varying degrees of drug tolerance/resistance [23, 26], indicating the existence of alternative signaling pathways that can bypass the intended intervention strategy. In this study, we have revealed a novel pathway in which PI3K phosphorylates ABHD17a to regulate Rap2b palmitoylation and control cell migration and invasion. This finding not only enhances our understanding of the complexity of EGFR signaling, but also provides a theoretical basis for the development of improved targeting strategies.

Rap2b belongs to the Ras superfamily, which is closely associated with tumorigenesis and cancer progression in humans [22, 27, 28]. Interestingly, multiple Ras proteins, such as H-/N-Ras, rely on a palmitoylation/depalmitoylation cycle to regulate their subcellular trafficking and oncogenicity [11, 22]. Specifically, palmitoylation often occurs on a cysteine residue adjacent to the CAAX motif at the C-terminus, as observed in H-Ras, N-Ras, and K-Ras4a [12, 29]. In accordance with this pattern, we have demonstrated that Rap2b is palmitoylated at C176/C177, which is two amino acids away from the CAAX motif (aa180-183) at the C-terminus (Fig. 1J). This suggests a potential pattern of S-palmitoylation for proteins containing the CAAX motif at the C-terminus. Furthermore, we have shown that ABHD17a is responsible for the depalmitoylation of Rap2b (Fig. 4), which aligns with the understanding that ABHD17a catalyzes PM-specific actions on dynamically palmitoylated proteins [30], considering that ABHD17a itself is also palmitoylated and localizes to the PM [30–32].

Although not identical, reversible palmitoylation controls the cycling of H-/N-Ras between the PM and the Golgi apparatus [11]. In the case of Rap2b, palmitoylation is essential for its localization to the PM, as inhibiting palmitoylation leads to the relocation of Rap2b from the PM to the cytosol (Figs. 2 and 4). Together, these findings reinforce the central concept that protein palmitoylation plays a crucial role in regulating membrane localization by increasing the hydrophobicity of modified proteins through the attachment of palmitate (a saturated fatty acid) to cysteine residues [15, 17, 33–38].

Although it is evident that palmitoyltransferases and depalmitoylases dynamically regulate the palmitoylation levels of their substrates, the mechanisms underlying the regulation of these enzyme activities remain unclear. Recent findings have shed light on this aspect. For instance, it has been discovered that AMPK phosphorylates ZDHHC13 to enhance its interaction with MC1R, resulting in increased MC1R palmitoylation and suppression of melanomagenesis [39]. Additionally, two separate studies have reported that LYN phosphorylates ZDHHC5 at Tyr91, leading to its deactivation in adipocytes [40], and that ZDHHC3 can be phosphorylated by FGFR (at Tyr18) and Src (at Tyr295 and Tyr297). Blocking these phosphorylation events increases ZDHHC3 autopalmitoylation and enhances NCAM palmitoylation in neurons [41]. Interestingly, in our study, we have revealed that the depalmitoylase ABHD17a is phosphorylated by PI3K at S302 and Y229. Disruption of these phosphorylation events further activates ABHD17a and reduces the level of palm-Rap2b. These examples highlight that the activities of enzymes involved in palmitoylation/depalmitoylation can be regulated through site-specific phosphorylation, making them potential critical molecular targets for therapeutic intervention.

It has been consistently reported that Rap2b is upregulated in various tumor types, including bladder cancer, lung cancer, hepatocellular carcinoma, and CRC [42–45]. Rap2b is known to modulate different aspects of cancer cells, such as cell adhesion, proliferation, migration, and invasion, through various signaling pathways, including PTEN/PI3K/AKT/ERK1/2, P53/PLC, and EGF/PLC/Src [7, 46–48]. In line with these findings, we have demonstrated that the levels of Rap2b and palm-Rap2b are significantly upregulated in CRC (Figs. 1A–E and 7A). We have shown that palm-Rap2b regulates migration and invasion, but not proliferation, in CRC cells through the EGF/PI3K/AKT signaling pathway (Figs. 3, 4, and 5C, D). Our findings have uncovered a previously unknown pathological mechanism in which Rap2b palmitoylation promotes cancer progression. Therefore, downregulating palm-Rap2b may have beneficial effects in CRC.

Different targeting strategies have been explored to downregulate Rap2b palmitoylation. One approach is to target the enzymes involved in depalmitoylation, such as ABHD17a and APT1, using enzymatic inhibitors [22, 30]. However, in our study, inhibiting ABHD17a to enhance palm-Rap2b is not aligned with the goal of downregulating Rap2b palmitoylation. Another approach is to use competitive peptides with the same protein sequence flanking the palmitoylation sites. However, the cellular treating effect of such peptides was not effective in our study (Fig. S7A), so this strategy was withdrawn. An alternative strategy is to directly block the palmitoylated peptide (C176/C177) at the C-terminus of Rap2b. Interestingly, we have designed a high-binding-affinity peptide named PTG-101, which significantly reduces the level of palm-Rap2b and suppresses cell migration and invasion both in vitro and in vivo (Figs. 7 and 8). This implies that palm-Rap2b is an important pharmaceutical target, and PTG-101 is an effective strategy for intervention.

In conclusion, our study has provided valuable insights into the post-translational modification landscape of Rap2b and its role in metastatic CRC. By targeting palm-Rap2b, we have identified PTG-101 as a potential therapeutic strategy for intervention. These findings contribute to the design of new therapies that specifically target metastasis in CRC and have the potential to improve patient outcomes.

## Materials and methods

### Animal care and human tissue samples

Balb/c or nude mice were obtained from Beijing Vital River Laboratory Animal Technology Co., Ltd. The mice were housed in a specific pathogen-free environment and kept in colony cages at a temperature of 25 °C, with a 12-h light and 12-h dark cycle, and provided with ad libitum access to food and water. CRC tissues and matched adjacent normal tissues were collected from patients who underwent surgery at the First Affiliated Hospital of Xinxiang Medical University, Xinxiang, China. All patients were not treated with adjuvant chemotherapy or radiotherapy before surgery. Primary tumor samples and adjacent tissues were subjected to IHC staining performed by the Department of Pathology of the First Affiliated Hospital of Xinxiang Medical University. The research was approved by the Ethics Committee of Xinxiang Medical University.

### Cell culture, transfection, and treatments

HEK293T cells (CRL-11268) were obtained from ATCC, while HCT-116 (TcHu133) and HCT-8 (TcHu 18) cells were purchased from the Cell Bank of the Chinese Academy of Sciences. All cell lines were cultured in DMEM high glucose medium (Gibco, USA) supplemented with 10% fetal bovine serum (Gibco, USA), and 100 μg/ml streptomycin and 100 U/ml penicillin, at 37 °C in a 5% CO_2_ incubator. SW480/SW620 cells (TCHu172/101, Cell Bank, Chinese Academy of Sciences) were cultured in Leibovitz’s L-15 Medium supplemented with 10% fetal bovine serum (Gibco, USA), and 100 μg/ml streptomycin and 100 U/ml penicillin, at 37 °C in a 100% air incubator. Transfections of all cell lines were performed using Lipofectamine 3000 reagent (Invitrogen) with the reduced serum medium Opti-MEM (Life Technologies), following the manufacturer’s instructions, for a duration of 24 h. The following reagents were used for cell treatments: 2-BP (Sigma-Aldrich, Cat#238422) and dimethyl sulfoxide (DMSO) (Sigma-Aldrich, Cat#D8418). Cells were treated when they reached 70–80% confluence.

### Plasmids

Plasmids expressing human-origin Flag-Rap2b (NM_002886.4), Flag-C176S-Rap2b, Flag-C177S-Rap2b, Rap2b-C176S/C177S-Flag, mouse-origin APT1-Flag, APT2-Flag, PPT1-Flag, PPT2-Flag, and ABHD17a-Flag (ABHD17a-S302A/-Y229A) were constructed. The cDNA for Rap2b, APT1, APT2, PPT1, PPT2, or ABHD17a was obtained by RT-PCR and then subcloned into pUC19 as cDNA donors. Specific primers were synthesized for subcloning individual cDNA into pCMV3-C-Flag for mammalian cell expression using the In-Fusion cloning method. All constructs were verified by sequence analysis.

### Acetyl-resin-assisted capture assay (Acyl-RAC assay)

The RAC assay was performed following previously described methods [18–20]. In brief, tissues or cells were collected and washed with cold PBS three times. They were then lysed in lysis buffer (20 mM Tris, pH 7.5, 150 mM NaCl, 1% Triton X-100) containing a protease inhibitor cocktail (Roche). The lysate was sonicated and incubated at 4 °C for 30 min with rotation. After centrifugation at 15,000 × *g* for 10 min at 4 °C, the supernatant was collected. Total protein was quantified using a bicinchononic acid assay Kit (Cat#P0009, Beyotime). To block thioester bonds, 1 mg of protein lysate was diluted to a concentration of 1 mg/ml in blocking buffer (100 mM HEPES, 1 mM EDTA, 2.5% SDS, 50 mM NEM, pH 7.5) and incubated at 50 °C for 60 min with shaking. NEM was removed by adding three volumes of cold acetone and performing four sequential 70% acetone precipitations. The resulting pellets were resuspended in 600 μl of binding buffer (100 mM HEPES, 1 mM EDTA, 1% SDS, pH 7.5). The samples were divided into two parts and 50 μl of prewashed thiopropyl Sepharose 6B (Cat#17-0420-01, GE Healthcare) was added to each part. To cleave thioester bonds, one part was added with 40 μl of 2 M hydroxylamine (HA) pH 7.0, while the other part was added with 40 μl of 2 M NaCl (negative control). Twenty microliters of each supernatant were collected as the “input” sample. Cleavage and capture were carried out on a rotator at room temperature for 4 h. The resins were washed five times with binding buffer. For western blot analysis, elution was performed by adding 40 μl of Laemmli loading buffer (2.1% SDS, 66 mM Tris-HCL, pH 7.5, 26% (W/V) glycerol, 50 mM 1,4-dithiothreitol (DTT)) and shaking at 42 °C for 15 min. The supernatants were collected, 1 μl of 5×SDS-PAGE loading buffer was added, and the samples were heated to 100 °C for 5 min before being analyzed by SDS-PAGE.

### Western blot analysis and antibodies

Samples were separated in standard SDS-PAGE gels and transferred to Immobilon-P PVDF membranes (pore size 0.2 μM, EMD Millipore). The membranes were then blocked in 5% (w/v) skimmed milk in TBS containing 0.1% (v/v) Tween-20 (TBST) for 90 min. After blocking, the membranes were washed in TBST and incubated with primary antibodies overnight at 4 °C. Following washing with TBST, the membranes were incubated with a suitable horseradish peroxidase (HRP)-labeled secondary antibody and signals were detected using an ECL kit (Tanon). The following primary antibodies were used: anti-Phospho-Ser/Thr (1:1000, ABclonal, Cat#AP1067)，anti-Flag (1:5000, ABclonal, Cat#AE092), anti-HA (1:5000, CST, Cat#2367S), anti-Rap2b (1:3000, Abcam, Cat#ab101369), anti-β-actin (1:20000, ABclonal, Cat#WH136110), anti-HSP90 (1:2000, CST, Cat#4874S), anti-Sodium potassium ATPase (1:3000, Abcam, Cat#ab76020), anti-AKT (1:2000, CST, Cat#4691S), anti-phospho-AKT (S473) (1:2000, CST, Cat#9271S), anti-GSK3β (1:2000, CST, Cat#12456S), anti-phospho-GSK3β (Ser9) (1:2000, CST, Cat#5558S), anti-MAPK (1:2000, CST, Cat#4685S), anti-phospho-MAPK (Thr202/Tyr199) (1:2000, CST, Cat#9101S), anti-NFκB (1:2000, CST, Cat#8242S), anti-phospho-NFκB (Ser536) (1:2000, CST, Cat#3033S), anti-E-Cadherin (1:1000, Santa Cruz, Cat#SC-8426), Goat Anti-Mouse IgG (1:5000, Protein Biotechnologies, Cat#PMS301), and Goat Anti-Rabbit IgG (1:5000, Protein Biotechnologies, Cat#PMS302). The following secondary antibodies were used for immunoblotting: Goat Anti-Mouse IgG (H + L), HRP Conjugate (Protein Biotechnologies, Cat#PMS301, 1:5000 for IB) and Goat Anti-Rabbit IgG (H + L), HRP Conjugate (Protein Biotechnologies, Cat#PMS302, 1:5000 for IB). β-actin was used as an internal reference for equal sample loading.

### mPEG-Mal labeling (mPEG)

The mPEG assay was performed as described [49]. A total of 500 μg of protein was treated with 10 mM tris(2-carboxyethyl)phosphine (TCEP) for 30 min and then incubated with 25 mM NEM for 2 h at room temperature to block unmodified cysteine residues. NEM blocking was terminated by three times methanol–chloroform–H_2_O precipitation (4/1.5/3) and the sample was dried in a speed-vacuum (Centrivap Concentrator, Labconco) after the last precipitation to remove NEM completely. The pellet was resuspended in 100 μl of TEA buffer (50 mM triethanolamine, TEA, 150 mM NaCl, 1×PI, pH 7.3) containing 4% SDS and incubated at 37 °C for 10 min with gentle agitation. Next, the samples were treated with 1 M neutralized NH_2_OH at room temperature for 1 h to cleave the thioester linkage formed between the cysteine residue and palmitate. The samples were then subjected to methanol–chloroform–H_2_O precipitation and resuspended in 30 μl of TEA buffer containing 4% SDS and 4 mM EDTA at 37 °C for dissolving. For mPEG-maleimide alkylation, an additional solution containing 0.2% Triton X-100, 90 μl of TEA buffer, and 1.33 mM mPEG-Mal (final concentration at 10 mM) was added and incubated at room temperature for 2 h. Finally, the reaction was stopped by a final methanol–chloroform–H_2_O precipitation and the sample was resuspended in Laemmli buffer (Bio-Rad) for western blot analysis.

### Protein purification for mass spectrometry

HEK293T cells were transfected with pCMV3-Flag-Rap2b using Lipofectamine 3000 as described above and harvested after 24 h with lysis buffer (20 mM Tris pH 7.5, 150 mM NaCl, 1% Triton X-100) containing protease inhibitor cocktail (Roche). The protein extract was clarified by centrifugation at 15,000 × *g* for 10 min at 4 °C twice. The Anti-DYKDDDDK Affinity resin (Sino Biological, Cat. No. 101274-MM13-RN) was equilibrated in equilibrating buffer (PBS 10 mM pH 7.4) three times and then the pre-cleared lysates were added and incubated at 4 °C with rotation overnight to pull down the Flag-Rap2b. The resin was pelleted by centrifugation and washed in equilibrating buffer (PBS 10 mM pH 7.4) three times. Elution was performed using eluting buffer (100 mM Glycine, 10 mM NaCl, pH 3.0). The resins were removed by centrifugation and the eluted supernatants were transferred to new tubes. The purified Flag-Rap2b was quantified using Coomassie blue staining (Beyotime, Cat#P0017).

### Mass spectrometric analysis of palmitoylation in Rap2b

Thirty micrograms purified Flag-Rap2b were digested using FASP [50]. Briefly, disulfide bonds were broken and blocked using 2 mM TCEP and 10 mM iodoacetamide (IAA), then proteins were transferred to 10 K filter, and cleaned sequentially using 8 M urea and 50 mM Tris-HCL pH 6.8 at 13,000 × *g*, 20 °C. GluC (P8100S, BioLabs) was added to filter at 1:50 (mass/mass) in 1x reaction buffer and proteins were digested at 37 °C for 16 h. Peptides were collected at 13,000 × *g*, 20 °C, lyophilized and stored at −80 °C until use. Raw files were acquired with data dependent acquisition mode using Orbitrap Fusion Lumos (San Jose, Thermo Fisher). Peptide mixture were separated on EasyNano LC1000 system (San Jose, Thermo Fisher) using both C18 (3 µm, 75 µm × 15 cm, homemade) and C4 column (5 µm, 75 µm × 15 cm, Thermo Fisher) at a flowrate of 600 nl/min. For peptide separation with C18 columns, a 60-min linear gradient was set as follows: 3% B (0.1% FA in ACN)/97% A (0.1% FA in H_2_O) to 8% B in 5 min, 8% B to 20% B in 38 min, 20% B to 30% B in 8 min, 30% B to 90% B in 2 min and stayed 7 min for 90% B. For peptide separation with C4 column, a 60-min linear gradient was set as follows: 3% B (0.1% FA in ACN)/97% A (0.1% FA in H_2_O) to 8% B in 5 min, 8% B to 20% B in 14 min, 20% B to 30% B in 18 min, 30% B to 90% B in 16 min and stayed 7 min for 90% B. For the data acquisition a top 20 scan mode with MS1 scan range m/z 350–1550 was used and other parameters were set as below: MS1 and MS2 resolution was set to 120 and 30 K; AGC for MS1 and MS2 was 4e5 and 1e5; isolation window was 1.6 Th, dynamic exclusion time was 15 s. To better identify modified amino acid sites, each precursor ion was fragmented with both HCD and EThcD. Collision energy of HCD was set to 32, and for EThcD collision energy was 25 and ETD reaction time was set automatically according to m/z and ion charge state of each precursor. Raw files were searched against target protein sequence using Byonic v2.16.11 (Protein Metrics). Searching parameter was set as follows: enzyme of GluC (semi) with maximum number of three missed cleavages; precursor and fragment ion mass tolerance was set to 20 and 50 ppm; variable modification was set to oxidation of M, deamidation of N, Q, carbamidomethylation of C, acetylation of protein N-term, palmitoylation of C, Y, S, T, W. An automatic score cut was used to remove low score peptides. A manual check was applied to further filter high-confident palmitoylated cysteine sites. Modified peptides only with continuous b and y product ions can be considered as a high confident modified site.

### Immunohistochemical (IHC) staining

Tumor tissues were fixed in formalin and embedded in paraffin. Representative tissue sections from the paraffin-embedded blocks were stained with primary antibodies and biotin-labeled anti-rabbit IgG for immunohistochemistry (IHC) analysis. A DAB detection kit was used to visualize the stained samples. The IHC results were quantified using a scoring system ranging from 0 to 12, as previously described [51]. A final score ≥4 was considered as “high expression”, while scores from 0 to 3 were defined as “low expression”.

### Immunofluorescence staining and imaging

Cells were seeded onto poly-d-lysine-coated coverslips and transfected with the indicated plasmids as described. After 24 h, the cells were fixed with 4% paraformaldehyde (Electron Microscopy Sciences, Cat#15710) and permeabilized with 0.1% Triton X-100 in PBS. Blocking was performed using 3% BSA in PBS. The cells were then stained with primary antibodies against Flag (Sigma, Cat#F7425, 1:500), HA (Sino Biological, Cat#10028-MM10, 1:500), Na/K-ATPase (Abcam, Cat# ab76020, 1:300), followed by incubation with secondary antibodies Anti-Rabbit IgG Alexa Fluor 488 (Thermo Fisher Scientific, Cat#A11034, 1:500) and Anti-Mouse IgG Alexa Fluor 594 (Thermo Fisher Scientific, Cat#A21135, 1:500). After washing, the cells were mounted onto slides using DAPI-Fluoromount-G (Electron Microscopy Sciences, Cat#17984-24). The fluorescence images were acquired using Stimulated Emission Depletion microscopy (Leica TCS SP8 STED).

### Cytosol and membrane protein fractionation

Cultured cells were harvested and washed three times with cold PBS. The cells were then suspended in 1 ml of HME buffer (10 mM HEPES, 1 mM MgCl_2_, 1 mM EDTA, pH 7.4) containing protease inhibitors. The cell lysates were subjected to six freeze/thaw cycles (freeze in liquid nitrogen and thaw at 37 °C). After sonication on ice, 150 μl of the lysate was taken as the “Total” sample. The remaining samples were briefly centrifuged at 500 × *g* to pellet intact cells and nuclei. The supernatants were carefully decanted and centrifuged at 20,000 × *g*, 4 °C for 1 h. The resulting pellet was resuspended in RIPA buffer (50 mM Tris, pH 7.4, 150 mM NaCl, 1% Triton X-100, 1% sodium deoxycholate, 0.1% SDS, 1 mM EDTA) containing protease inhibitors, and designated as the membrane fraction. The supernatant was collected as the cytosolic fraction. The protein concentrations were quantified and analyzed by western blotting.

### Deleting Rap2b in HCT-116 cells line

The online tool (http://crispor.tefor.net) was used to design sgRNA targeting the exon of RAP2B (Gene ID: ENST00000323534.4). For cloning purposes, a BbsI restriction site was added. The sgRNAs were synthesized by Shanghai Bioligo Biotechnology Co., Ltd. (Shanghai, China). The Px458 vector was digested with BbsI (NEB #R3539), annealed with the sgRNA, and then transferred into DH5α competent cells (Beijing Protein Biotechnology Co., Ltd., Beijing, China) for transformation. The positive clones (ampicillin-resistant) were screened and sequenced by Wuhan Genecreate Bioengineering Co., Ltd. (Wuhan, China). The recombinant plasmid was extracted using a Tiangen kit (Tiangen Biotechnology Co., Ltd., Beijing, China) for max-preparation. HCT-116 cells were prepared and transfected with the two pX458 vectors using Lipofectamine™ 3000 Transfection Reagent (Invitrogen). After 48 h, single fluorescent cells were sorted into a 96-well plate using FACS (BD Biosciences, San Jose, CA). After ~2 weeks, single cell colonies were obtained. DNA was extracted from these cells and subjected to PCR amplification and sequencing to identify positive clones. The PCR reaction conditions were as follows: 35 cycles including 94 °C for 2 min, 94 °C for 30 s, 55 °C for 30 s, and 72 °C for 30 s, followed by a final extension at 72 °C for 2 min.

### Mass spectrometric analysis of ABHD17a phosphorylation

Briefly, ABHD17a protein lanes were cut into pieces about 1 mm^3^. Gel pieces were washed with distilled water followed by 50% acetonitrile (ACN)/100 mM NH_4_HCO_3_ (pH 8.0) for three times and then incubated with 100% ACN. After the incubation, samples were reduced with 10 mM DTT at 65 °C for 1 h followed by alkylation with 55 mM IAA at room temperature in the dark for 30 min. Finally, gel pieces were washed with 100% ACN and dried in a vacuum concentrator. Overnight tryptic digestion was conducted in 50 mM NH_4_HCO_3_ at 37 °C. After in-gel digestion, the peptides were extracted with 60% ACN/5% formic acid aided with ultrasonic bath. Collected peptide samples were vacuum dried and purified using C18 desalting columns. The eluate was vacuum dried and stored at −20 °C for later use.

MS analysis was performed on a trapped ion mobility quadrupole time-of-flight mass spectrometer timsTOF Pro (Bruker Daltonics). An UltiMate 3000 RSLCnano system (Thermo) was coupled online to the timsTOF Pro with a CaptiveSpray nano ion source (Bruker Daltonics). Peptide samples were injected onto a Trap column (75 μm × 20 mm, 2 μm particle size, 100 Å pore size, Thermo), and separated on a reversed-phase C18 analytical column (75 μm × 250 mm, 1.6 μm particle size, 100 Å pore size, IonOpticks). Mobile phase A (0.1% formic acid in H_2_O) and mobile phase B (0.1% formic acid in ACN) were used to establish the separation gradient. Liquid chromatography was performed with a constant flow of 300 nL/min. The MS was operated in diaPASEF (parallel accumulation-serial fragmentation) mode with TIMS on. Each cycle comprised one full MS1 scan and followed MS/MS scans. MS raw data were analyzed with MaxQuant (V2.0.1) using the Andromeda database search algorithm. Spectra files were searched against the SwissProt Mouse protein database using the following parameters: variable modifications, phosphorylation (STY), fixed modifications, carbamidomethyl (C); digestion, trypsin/P. Search results were filtered with 1% FDR at both protein and peptide levels. Proteins denoted as decoy hits, contaminants, or only identified by sites were removed, the remaining identifications were used for further quantification analysis.

### Wound-healing assay

Wound-healing assays were carried out using the ibidi Culture-Insert 2 Well, following the manufacturer’s instructions (Ibidi, 80206, 35 mm). Briefly, 1 × 10^5^ cells in 100 μl were seeded in each well to ensure confluency within 24 h. The cells were then incubated at 37 °C and 5% CO_2_ for 24 h. Afterward, the Culture-Insert 2 Well was removed using sterile tweezers, and the well was filled with fresh serum-free DMEM medium. The cells were incubated for an additional 48 h, followed by three washes with PBS. Images were captured at 0, 24, or 48/72 h after creating the wound. The motility of the cells was assessed by measuring the distance/percentage between the wound edges.

### Migration and invasion assay

Cell migration and invasion assays were conducted using Transwell units with 6.5 µm-pore polycarbonate filters (Corning Incorporated, Corning, NY, USA). For the migration assay, cells cultured in 150 μl DMEM were seeded in the upper chamber, while 500 μl medium with 20% FBS was added to the lower chamber. The cells were then incubated for 24 h, fixed, and stained with crystal violet. Cells in the upper chamber were removed using a cotton swab, and the number of cells that had migrated across the membrane was counted in five randomly selected microscopic fields. For the invasion assay, a similar procedure was followed, but Matrigel-coated chambers (BD Biosciences) were used instead of Transwell chambers. After 48 h of incubation at 37 °C, the cells that had invaded into the bottom well were quantified. All experiments were independently repeated at least three times for statistical analysis.

### Virus packaging in HEK293T cell

The lentivirus was packaged in HEK293T cells using a previously described method [51]. In brief, 20 μg of the recombinant plasmid was transfected into HEK293T cells along with 15 μg of helper plasmids (pSPAX2 and pMD2.G). After 36 h of transfection, the medium containing lentiviral particles was collected. The lentiviral particles were either concentrated at 70,000 × *g* for 2 h or directly aliquoted and stored at −80 °C until further use.

### The construction of HCT-116 cells line stably expressing firefly luciferase

Rap2b-KO cells were transfected with the Luciferase-mCherry-puro virus obtained from Hanbio Aden Vector Institute (Shanghai, China). mCherry-positive cell clones, referred to as Rap2b-KO^luc^, were selected through serial dilution and confirmed using western blot and immunofluorescence microscopy. The bioluminescence of the cells was confirmed using the PekinElmer IVIS Spectrum CT imaging system. Subsequently, the Rap2b-KO^luc^ cells were infected with lentivirus encoding either GFP-Rap2b or GFP-Rap2b-2CS. The infection was performed in the presence of 8–10 μg/ml polybrene in the culture medium. GFP-positive cell clones were selected through serial dilution and confirmed using western blot and immunofluorescence microscopy.

### A designed peptide targeting RAP2B palmitoylation

For blocking the active site of Rap2b, we first sought for natural derived sequence that targeting motif NH–GCCS-CO (aa175-178). This sequence could also be found in some other proteins but all the conformation of motif NH–GCCS-CO displayed a random coil according to co-crystal structures, which was tricky for peptide design (PDBID: 2MD6, 4E6K, and 6E6S). Considering the structural similarity between cysteine and serine, we focused our attention on motif NH–EGSSSA-CO. In a crystal structure of isopullulanase (PDBID: 1X0C), motif NH–EGSSSA-CO formed a β-sheet structure with motif NH–ESFEPLSINTTE-CO [52]. But a part in this motif, NH-PLSINT-CO, formed a small coil and seemed to have no contribution to peptide binding. Hence, we simplified this peptide to H_2_N–ESFEXTE-COOH and further introduced β-ala to this peptide as a linker. The resulted peptide H_2_N–ESFEβATE-COOH (PTG-101) was proved to be able to mimic the conformation of NH–ESFEPLSINTTE-CO in the subsequent molecular dynamic simulation (Fig. S6A, B).

### Xenograft model via tail vein injection

Balb/c nude mice (4–5 weeks old, 8 males/group) received HCT-116 tumor cells (100 µl of 1 × 10^6^ cells/ml in PBS) through the dorsal tail vein. Prior to injection, warm the animal for 5–10 min to dilate the veins. The mice were immobilized using a mouse restraint device. Ensure correct orientation and location of the lateral tail vein before performing injections. Keep the needle and syringe parallel to the tail. When positioned correctly, the needle should smoothly enter the vein. Slowly inject the cells; you should encounter no resistance when depressing the plunger. The establishment of the xenograft model is confirmed by the positive detection of bioluminescence in lung (10 days after injection) using the PerkinElmer IVIS Spectrum CT imaging system. Subsequently, the animals were randomly allocated to different experimental groups.

### Peptide preparation and treatment in xenograft mouse Model

PTG-101 was packed in liposome for treatment. To prepare PTG-101-LNPs, Chol and PL-100M (20.5 mg) with the ratio of 1:9 (w/w) were dissolved in chloroform, and evaporated under reduced pressure to obtain a lipid film. Thereafter, 1 mL physiological saline solution with 0.64 mg/ml PTG-101 was added, incubated at 20 °C for 10 min. The obtained preparation was extruded thrice through a 200 nm polycarbonate membrane. PTG-101 was given 10 days after tumor inoculation by tail vein injection, 0.25 ml/20 g mouse/daily. All animal procedures were performed according to guidelines approved by the Committee on Animal Care at Xinxiang Medical University.

### 3D cell culture for HCT-116 cells

HCT-116 cells were used for 3D culture, the cells were trypsinized and resuspended at a concentration of 10^4^ cells/ml in media supplemented with 2% Matrigel (356234, BD). Next, 1 ml of cell suspension was plated into each 15 mm glass-bottom cell culture dish (801002, Nest) previously coated with 80 μl of Matrigel. The cells were allowed to grow for 48 h, with the medium replaced every 48 h with fresh medium containing 2% Matrigel over a period of 10–14 days. The formation of HCT-116 cysts was monitored using a phase-contrast microscope.

### Data acquisition and analysis

RNA sequencing data and corresponding clinical information for the GDC TCGA Colon Cancer (COAD) cohort were downloaded from the UCSC Xena platform (https://xena.ucsc.edu/). RNA sequencing data were batch-corrected and normalized (log2(fpkm + 1)). Clinical information included patient survival and outcomes. Using the CMS_network prediction method described in the paper “The consensus molecular subtypes of colorectal cancer”, CRC samples were classified into four consensus molecular subtypes (CMS). Sample IDs for each CMS group were obtained from the Colorectal Cancer Subtyping Consortium (CRCSC) (https://www.synapse.org/Synapse:syn2623706/wiki/67246). A total of 473 cancer samples from TCGA-COAD were initially included. After screening, 328 samples were included for further analysis, consisting of 69 CMS1, 131 CMS2, 48 CMS3, and 80 CMS4 samples. RNA sequencing data were normalized and converted to log2(TPM + 1) format. The expression levels of the RAP2B gene among the four CMS subtypes in COAD samples were compared using the ggplot2 package (v3.5.1) in R to generate box plots. ANOVA for multiple comparisons was used to calculate the global *p* value, and *t*-tests were performed to compare each group to all groups (base mean). To assess the impact of RAP2B gene expression levels on patient survival, survival analyses were performed using the survival package (v3.7-0) and the survminer package (v0.4.9). The optimal cutoff value was determined using the surv_cutpoint function, dividing patients into high and low RAP2B expression groups within each CMS subtype. Kaplan–Meier survival curves were plotted and log-rank tests were used to compare survival differences between groups.

### Statistical analysis

Descriptive data were expressed as means ± standard errors of means. Statistical analysis of differences between two groups was performed using paired or unpaired two-tailed Student’s *t*-tests when applicable. In the case of unequal variance between the two groups, the unpaired Welch’s *t*-test was used. For experiments with more than two groups, one-way ANOVA analysis was conducted. Dunnett T3’s post hoc test (data with unequal variance) or by Tukey’s post hoc test (data with equal variance) were performed when necessary. An alpha level of 0.05 was considered statistically significant for all analyses. Graphs were generated using GraphPad Prism software for Windows, version 5.

## Supplementary information

Supplemental figure legends

Supplemental figures

Supplemental figure 1-uncropped western blots

Supplemental tables

## Data Availability

The published article includes all data sets generated/analyzed for this study. Additional data are available from the corresponding author on reasonable request., EYK (eykong2012@163.com).

## Materials availability

Requests for materials and reagents should be directed to and will be fulfilled by the lead Contact with acompleted Materials Transfer Agreement., EYK (eykong2012@163.com).
