# Supplementary material for: Inhibiting S-palmitoylation arrests metastasis by relocating Rap2b from plasma membrane in colorectal cancer
Source: Cell Death Dis. 2024 Sep 14;15(9):675. doi: 10.1038/s41419-024-07061-2 (PMC11401852; doi:10.1038/s41419-024-07061-2)
Supplement: Supplementary file 1 — Supplemental figure legends [file 41419_2024_7061_MOESM1_ESM.docx]

**Supplemental figure legends**

**Fig. S1. Identification of palmitoylation of purified Rap2b using mass spectrometry.**

(A) Flag-Rap2b was immunoprecipitated using Flag-agarose beads and eluted for Coomassie blue staining.

(B-C) The eluted immunoprecipitate was analyzed by mass spectrometry. Sites of interest were quantified for protein palmitoylation (B), and the peptide coverage of Rap2b was illustrated (C).

(D) HCT-116 cells expressing Flag-Rap2b were subjected to mPEG-labeling assay and analyzed by Western blot.

**Fig. S2. Rap2b palmitoylation is necessary for its localization to the plasma membrane in HCT-8 cells.**

(A-B) HCT-8 cells were transfected with Rap2b and its mutant forms for immunofluorescence analysis (A), and the distribution of their proteins was profiled (B).

**Fig. S3. Deletion of Rap2b in HCT-116 cells.**

(A) Schematic representation of the targeting strategy to truncate the exon in human Rap2b. Two sgRNAs were designed to target the sequences CGCGCTCGTACCGCTTCACGCGG and GTGAGCGCGAGGTCTCGTACGGG in human Rap2b.

(B) Sequencing confirmed a deletion of 73 bp in the knockout allele.

(C) The knockout allele is predicted to result in a frameshift mutation in Rap2b based on ORF finder analysis.

(D) Genotyping was performed by PCR amplification using specific primers (F-CGTCGGTGCTGGAGATCC, R-TAGTTCATCTGCCGCACGAT), resulting in amplicons of 343 bp and 270 bp for the wildtype and knockout alleles, respectively.

(E) Western blot confirmed the deletion of Rap2b in the Rap2b-KO cells.

**Fig. S4. Rap2b modulates cell proliferation and migration in HCT-8 cells.**

(A) CCK-8 assay was performed to analyze the proliferation of wildtype (WT) and Rap2b-KO HCT-116 cells.

(B) Overexpression of Rap2b in HCT-116 cells was assessed using the CCK-8 assay. (C-F) HCT-8 cells expressing Rap2b and Rap2b-2CS were evaluated using wound-healing assays (C-D) and Matrigel invasion assays (E-F).

**Fig. S5. ABHD17a regulates the membrane localization of Rap2b in HCT-8 cells.**

(A) Immunofluorescence analysis was performed on HCT-8 cells expressing Rap2b and/or ABHD17a.

(B) Cytosol/membrane fractionations were carried out on HCT-8 cells expressing Rap2b and/or ABHD17a, and the results were analyzed by Western blot and quantified. ***P<0.001, 2-tailed t-test, n=3 biological replicates. Data are presented as mean ± S.E.M.

**Fig. S6. Strategic design and predicted docking of PTG-101.**

(A) Molecular dynamic simulation showed that the peptide H2N–ESFEβATE-COOH (PTG-101, green) mimics the conformation of NH-ESFEPLSINTTE-CO (blue).

(B) Predicted docking mode of PTG-101 (green) with the NH–EGSSSA-CO motif (aa469-474) in the crystal structure of isopullulanase (PDBID: 1X0C). Hydrogen bonds are shown as blue dashed lines.

**Fig. S7. PTG-101 downregulates palm-Rap2b, alters its plasma membrane localization, and affects migration in HCT-8 cells.**

(A) Acyl-RAC analysis was performed on wildtype (WT) HCT-116 cells treated with either DMSO or various peptides to assess palm-Rap2b levels.

(B) Acyl-RAC analysis was performed on WT HCT-116 cells treated with different dosages of PTG-101 or transfected with ABHD17a to examine palm-Rap2b levels.

(C-D) HCT-8 cells treated with or without PTG-101 were subjected to Acyl-RAC analysis to evaluate palm-Rap2b levels (C), and the results were quantified (D). **P<0.01, 2-tailed t-test, n=5 biological replicates.

(E) Immunofluorescence imaging was performed on HCT-8 cells expressing Flag-Rap2b, treated with or without PTG-101.

(F) Subcellular fractionation and Western blot analysis were carried out on HCT-8 cells expressing Flag-Rap2b, treated with or without PTG-101, and the results were quantified. ****P<0.0001, 2-tailed t-test, n=3 biological replicates.

(G-H) Wound-healing assays were performed on HCT-8 cells incubated with or without PTG-101, and the percentage of wound width was quantified at different timepoints. ***P<0.001, ****P<0.0001, 2-tailed t-test, n=3 biological replicates. Data are presented as mean ± S.E.M.

**Fig. S8. Rap2b is palmitoylated in SW480 and SW620 cells, and inhibiting Rap2b palmitoylation with PTG-101 alters its subcellular localization and cellular migration.**

(A) The protein levels of Rap2b were evaluated in SW480 and SW620 cells by WB. Statistical analysis was conducted using a two-tailed t-test. **P<0.01, n=3 biological replicates.

(B) The levels of Rap2b palmitoylation were examined in SW480 and SW620 cells by RAC assay. Statistical analysis was performed using a two-tailed t-test. ***P<0.001. n=3 biological replicates.

(C-D) SW480 cells, treated with or without PTG-101, were evaluated for Rap2b palmitoylation levels by RAC assay and quantified. Statistical analysis was performed using a two-tailed t-test. **P<0.01. n=3 biological replicates.

(E-F) SW620 cells, treated with or without PTG-101, were evaluated for Rap2b palmitoylation levels by RAC assay and quantified. Statistical analysis was performed using a two-tailed t-test. **P<0.01. n=3 biological replicates.

(G) SW480/SW620 cells expressing either Flag-Rap2b (treated with or without PTG-101) or Flag-Rap2b-2CS were subjected to immunofluorescence analysis.

(H-I) Cytosol/membrane fractionation and Western blot analysis of SW480/SW620 cells treated with or without PTG-101. Statistical analysis was performed using a two-tailed t-test. ***P<0.001, ****P<0.0001. n=3 biological replicates.

(J) Wound healing assay of SW480 cells treated with or without PTG-101. Statistical analysis was performed using a two-tailed t-test. ****P<0.0001. n=3 biological replicates.

(K) Transwell and Matrigel invasion analysis of SW480 cells treated with or without PTG-101. Statistical analysis was performed using a two-tailed t-test. ****P<0.0001. n=3 biological replicates.

(L) Wound healing assay of SW620 cells treated with or without PTG-101. Statistical analysis was performed using a two-tailed t-test. ****P<0.0001. n=3 biological replicates.

(M) Transwell and Matrigel invasion analysis of SW620 cells treated with or without PTG-101. Statistical analysis was performed using a two-tailed t-test. ****P<0.0001. n=3 biological replicates. Data are presented as mean ± S.E.M.

**Fig. S9. Rap2b is differentially expressed and correlated with the survival of CRC patients.**

(A) The expression levels of Rap2b in CMS1-4 categories of CRC patients (TCGA dataset). t-tests were performed to compare each group to all groups (base mean). CMS1 (n=69), CMS2 (n=131), CMS3 (n=48), and CMS4 (n=80). ****P<0.0001, *P<0.05, n.s. indicates not significant.

(B) paraffin sections of WT mouse colon were immunostained with Rap2b antibody.

(C) The impact of RAP2b expression levels on CRC patient survival.

(D) SW480/SW620 cells were used for the wound healing assay, and cells from varied localization were collected for the evaluation of Rap2b expression. Statistical analysis was performed using a two-tailed t-test. **P<0.01. n=3 biological replicates.

(E) HCT-116 cells were seeded in matrigels for 3D cell culture, and cells from varied localization were collected for the evaluation of Rap2b expression. Statistical analysis was performed using a two-tailed t-test. **P<0.01. n=3 biological replicates. Data are presented as mean ± S.E.M.

**Fig. S10.** Uncropped blots for Fig. 1.

**Fig. S11.** Uncropped blots for Fig. 2.

**Fig. S12.** Uncropped blots for Fig. 4.

**Fig. S13.** Uncropped blots for Fig. 5.

**Fig. S14.** Uncropped blots for Fig. 6.

**Fig. S15.** Uncropped blots for Fig. 7.

**Fig. S16.** Uncropped blots for Fig. 8.
