# Supplementary figures and images for: Inhibiting S-palmitoylation arrests metastasis by relocating Rap2b from plasma membrane in colorectal cancer
Source: Cell Death Dis. 2024 Sep 14;15(9):675. doi: 10.1038/s41419-024-07061-2 (PMC11401852; doi:10.1038/s41419-024-07061-2)

Fig. S1

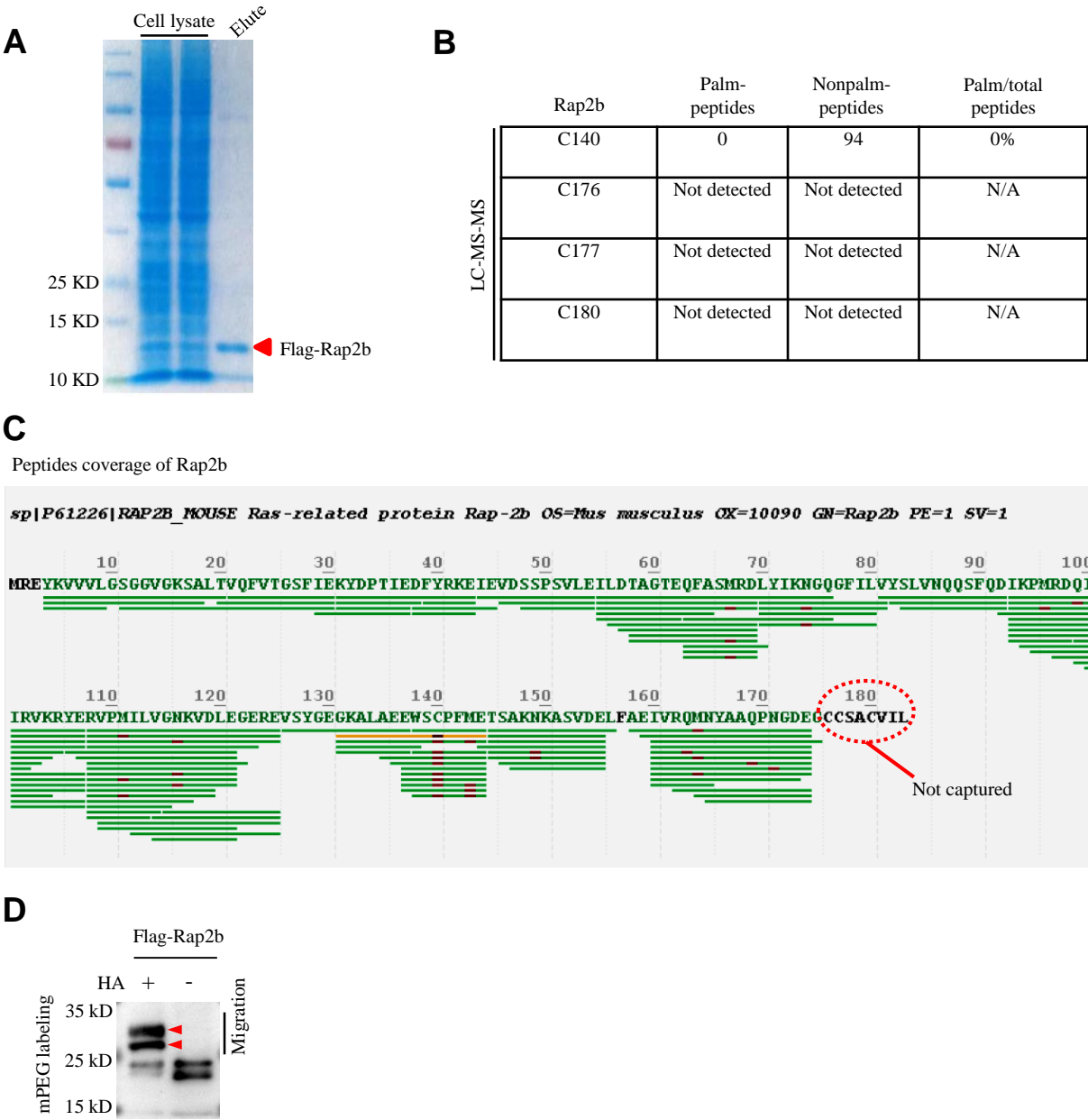

Fig. S2

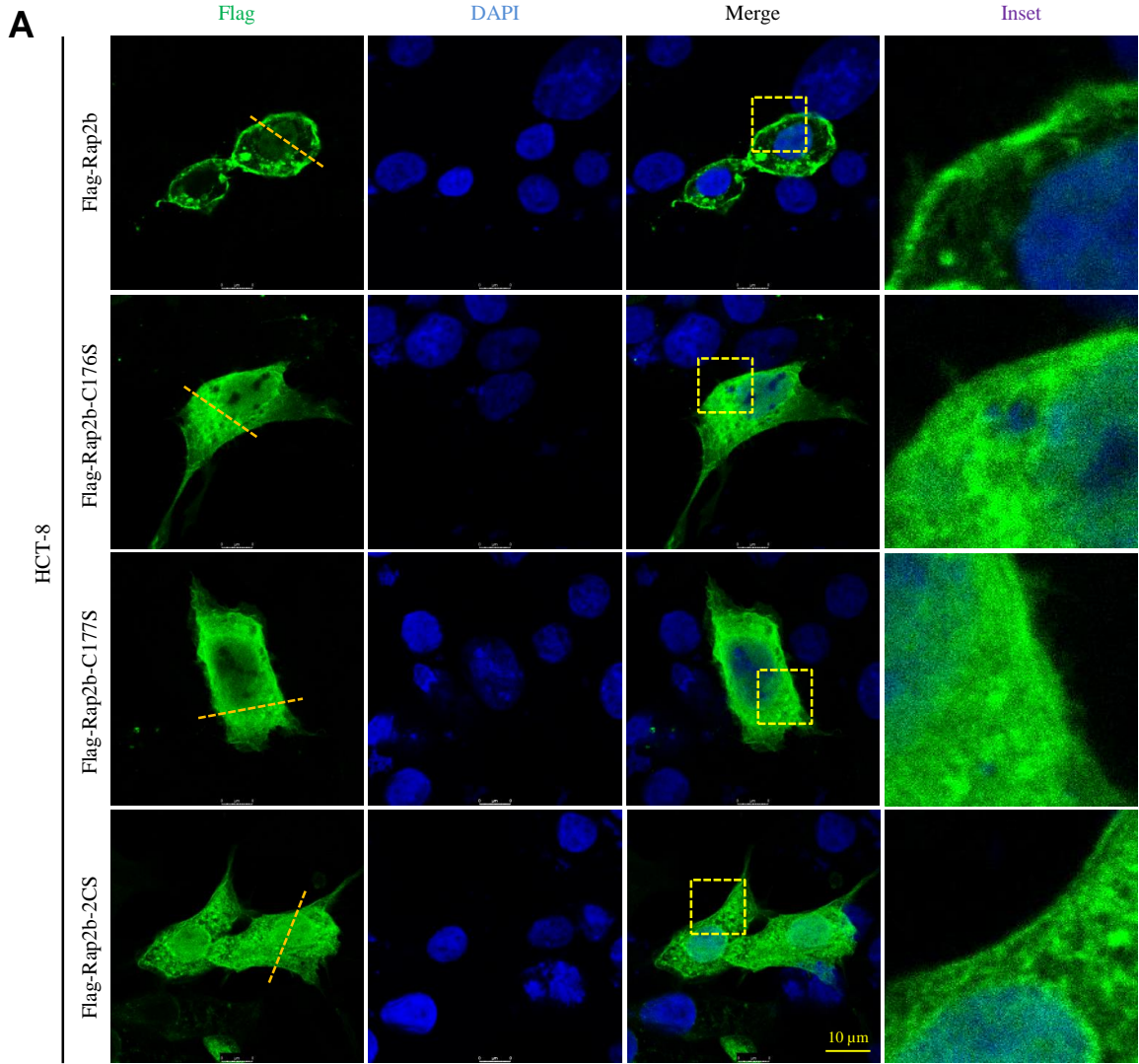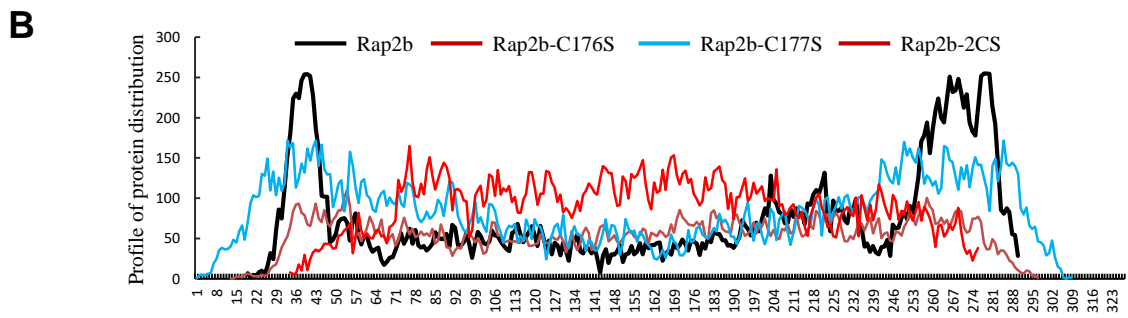

Fig. S3

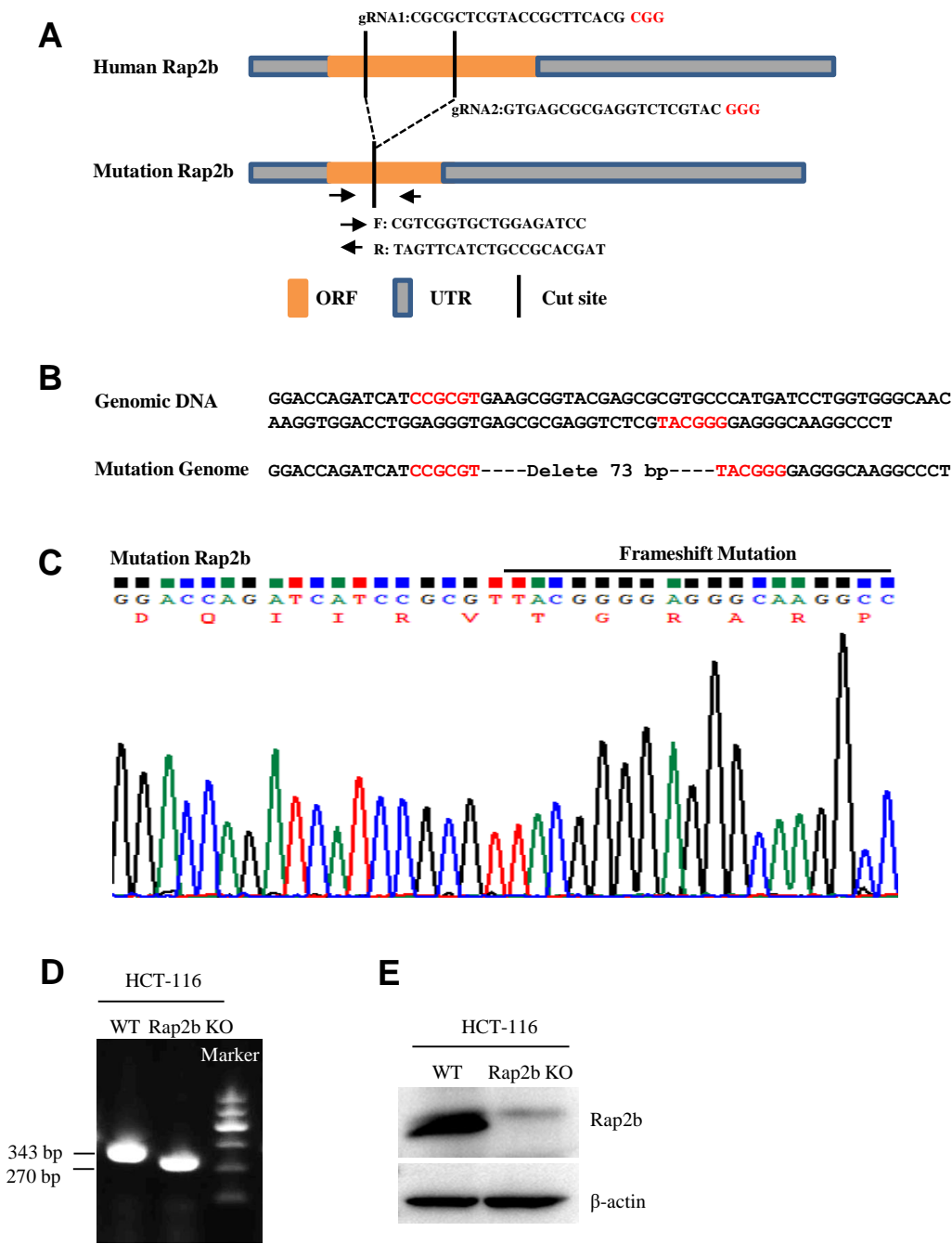

Fig. S4

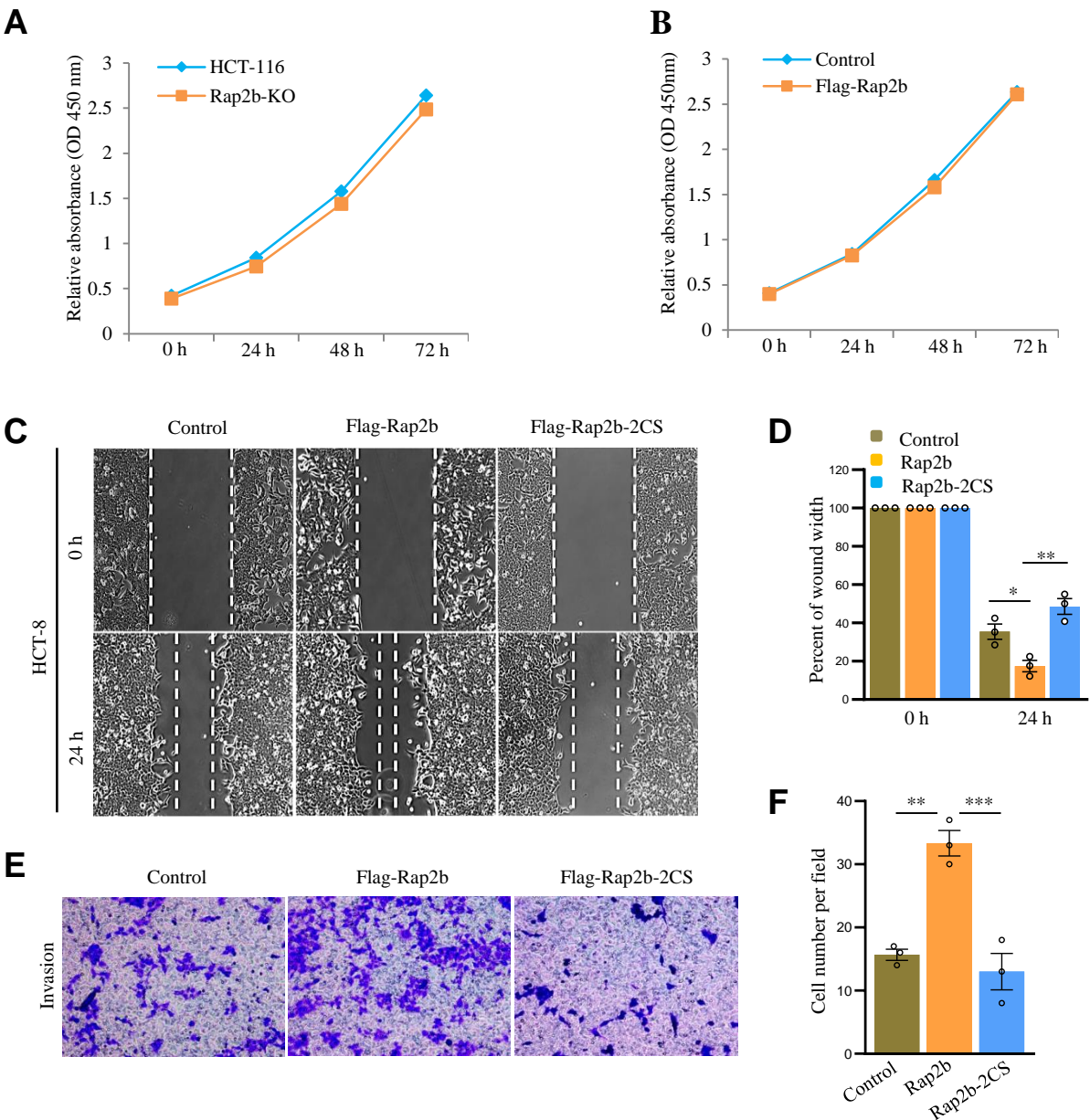

Fig. S5

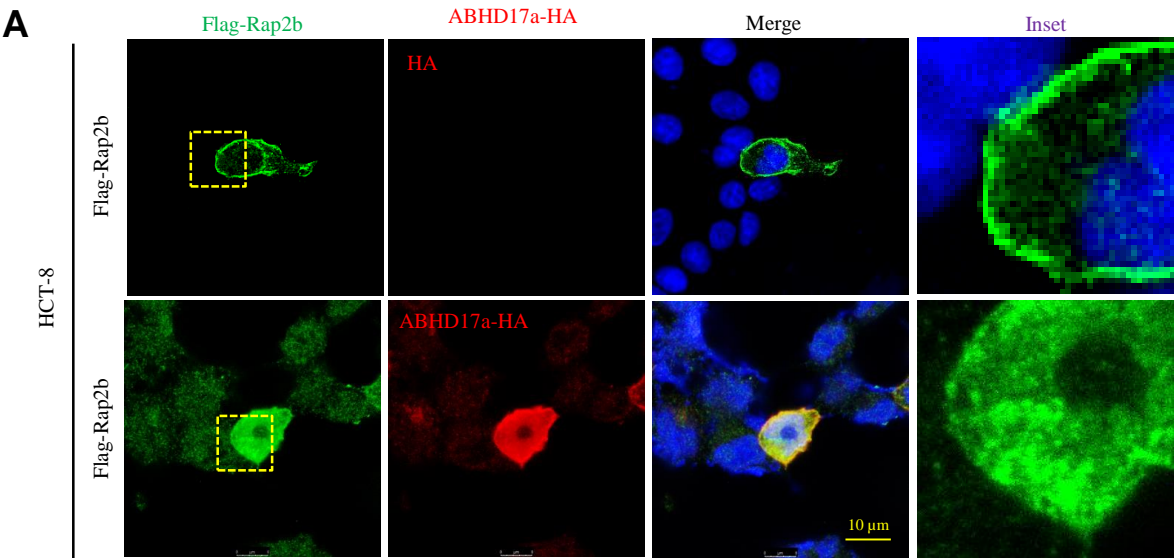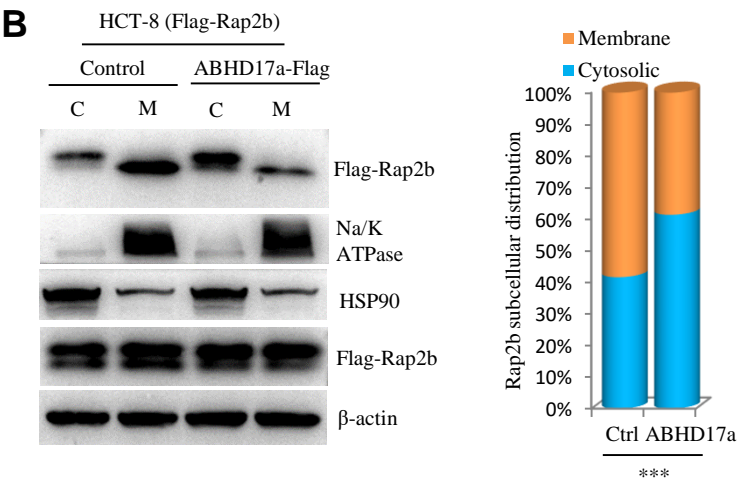

Fig. S6

**A**

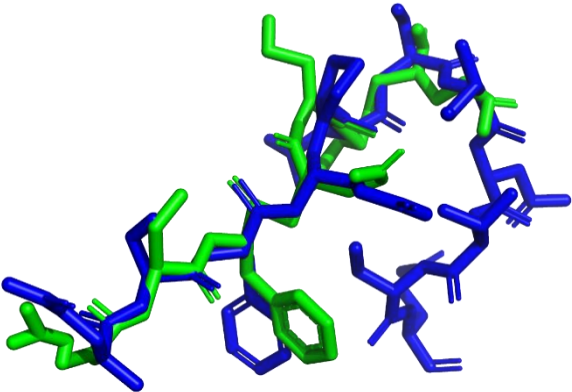

**B**

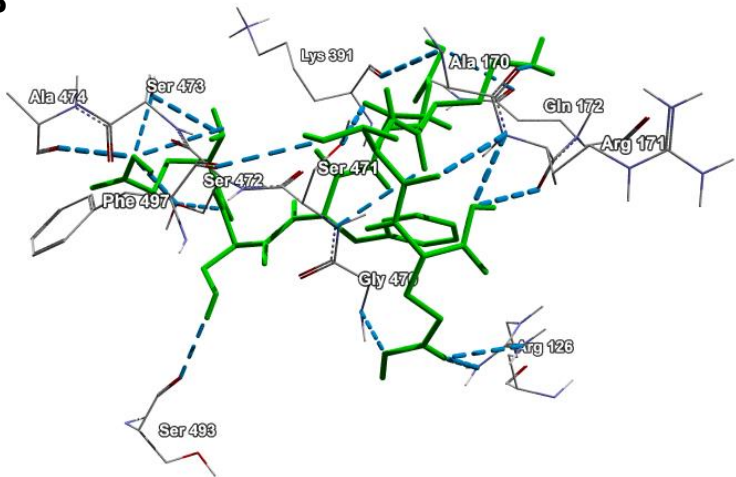

Fig. S7

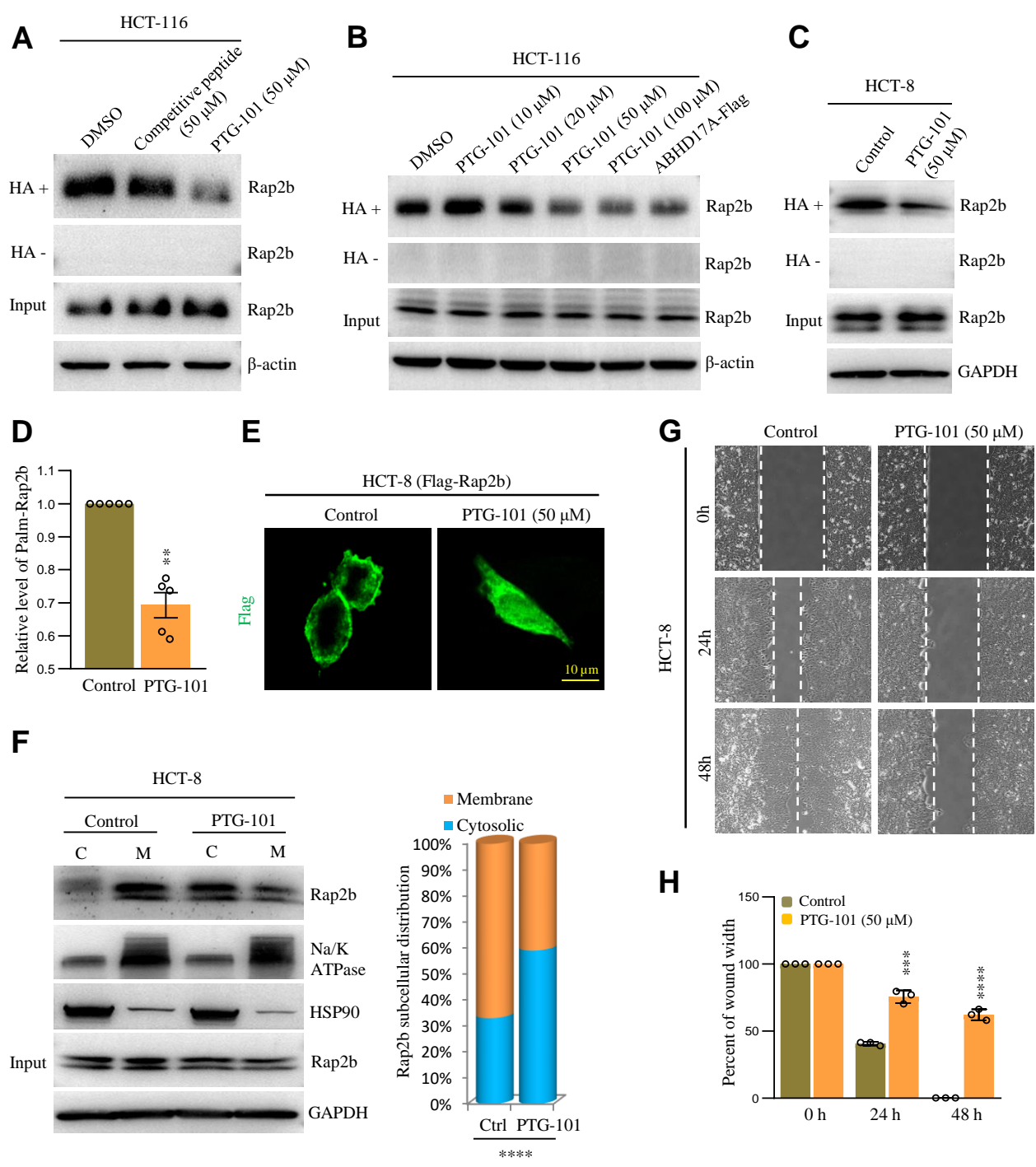

Fig. S8

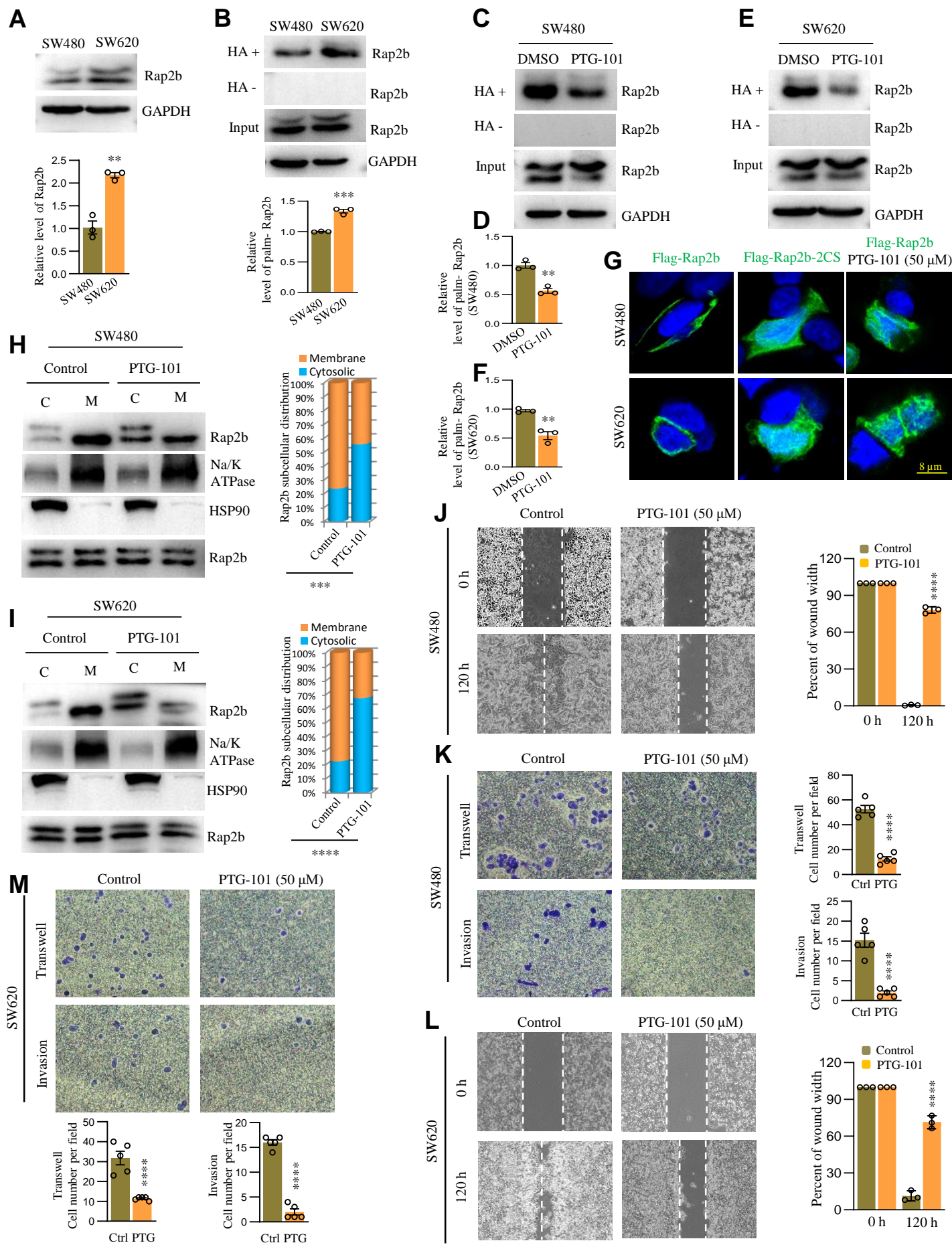

Fig. S9

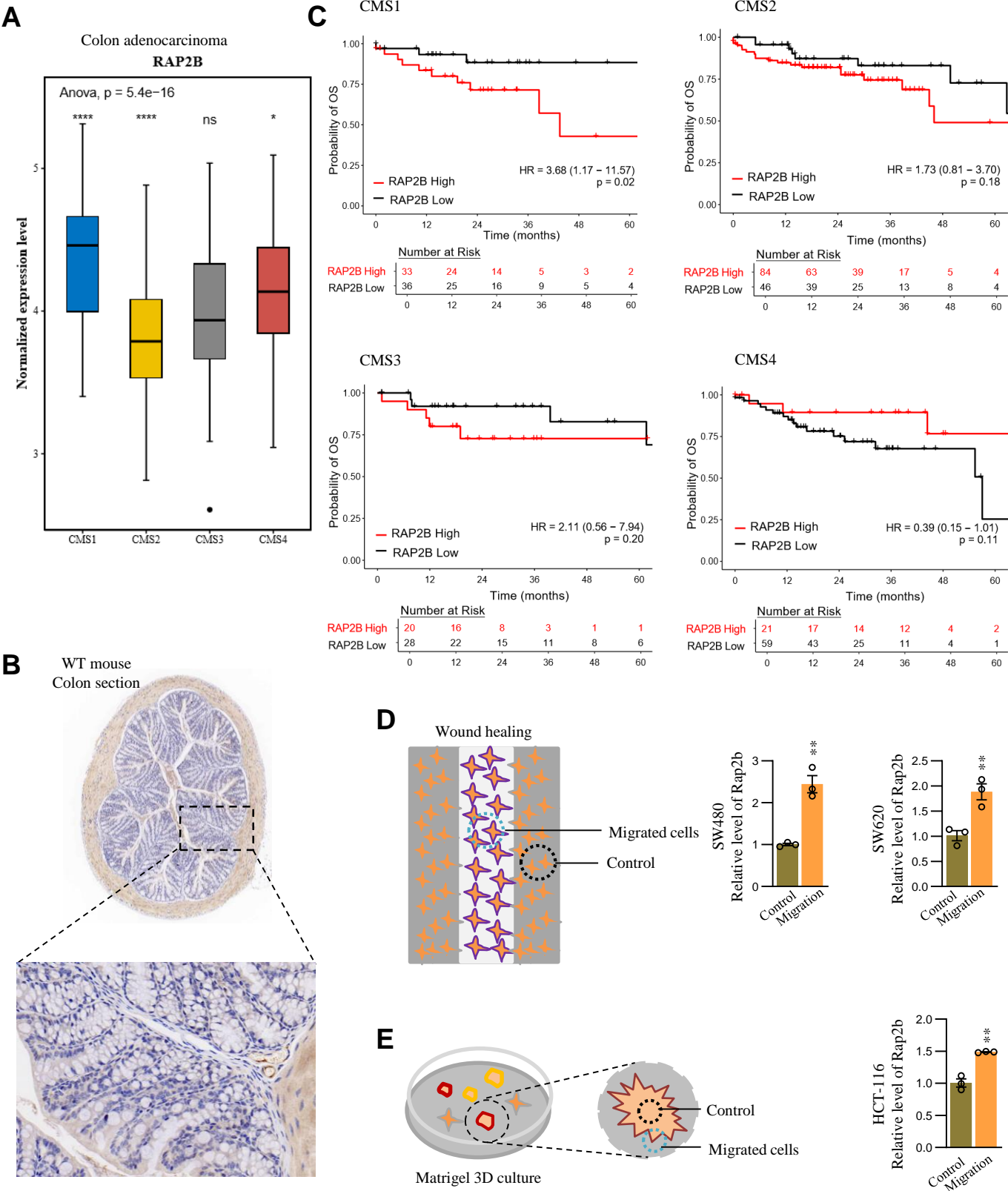

Supplement: Supplementary file 2 — Supplemental figures [file 41419_2024_7061_MOESM2_ESM.pdf]
