# Supplementary material for: Inhibiting S-palmitoylation arrests metastasis by relocating Rap2b from plasma membrane in colorectal cancer
Source: Cell Death Dis. 2024 Sep 14;15(9):675. doi: 10.1038/s41419-024-07061-2 (PMC11401852; doi:10.1038/s41419-024-07061-2)

Fig. S10

Uncropped blot of Fig.1F

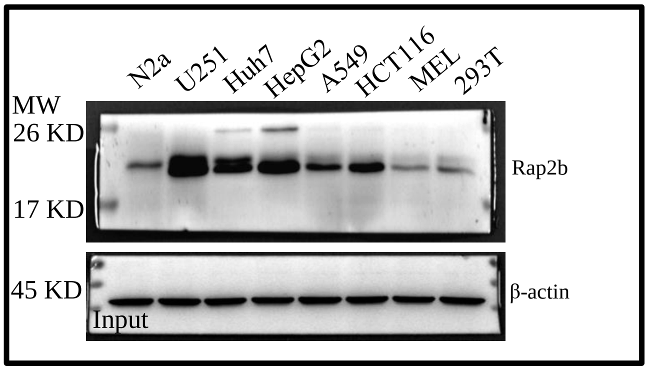

Uncropped blot of Fig.1G-1H

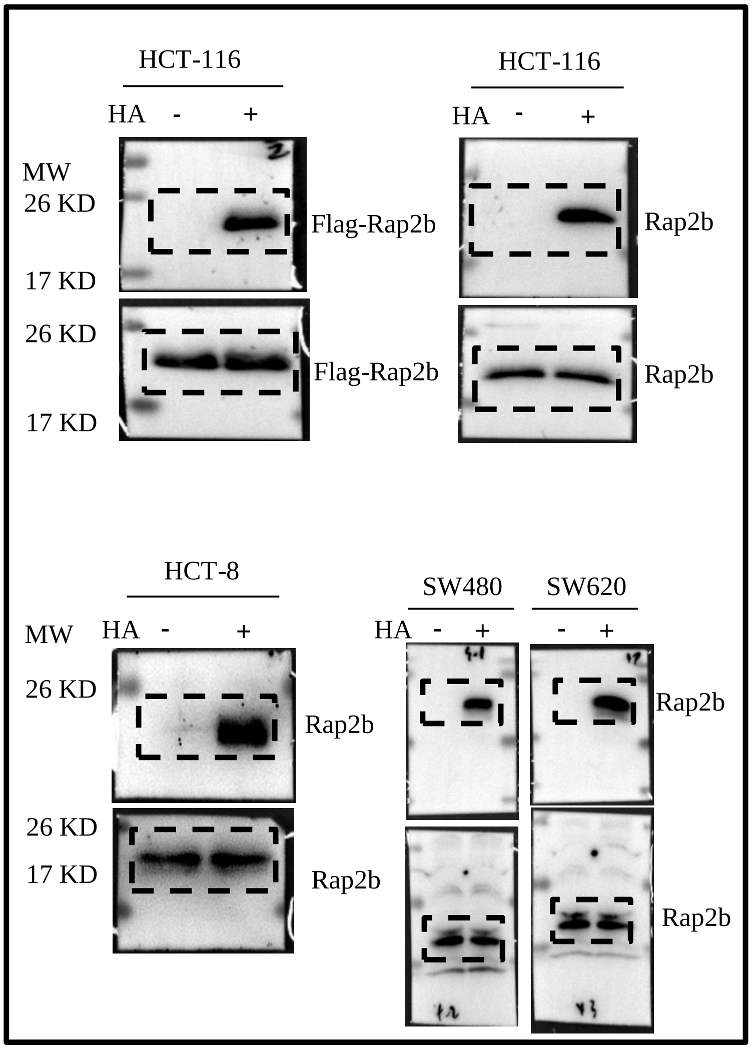

Uncropped blot of Fig.1K

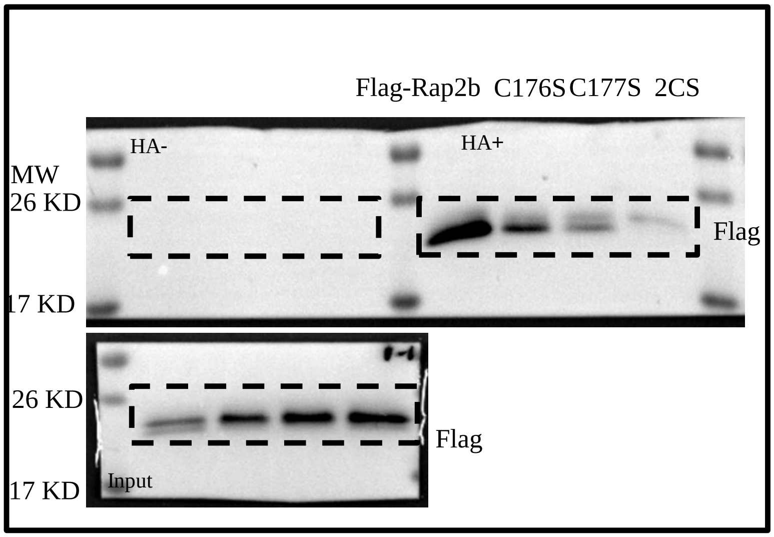

Uncropped blot of Fig.1I

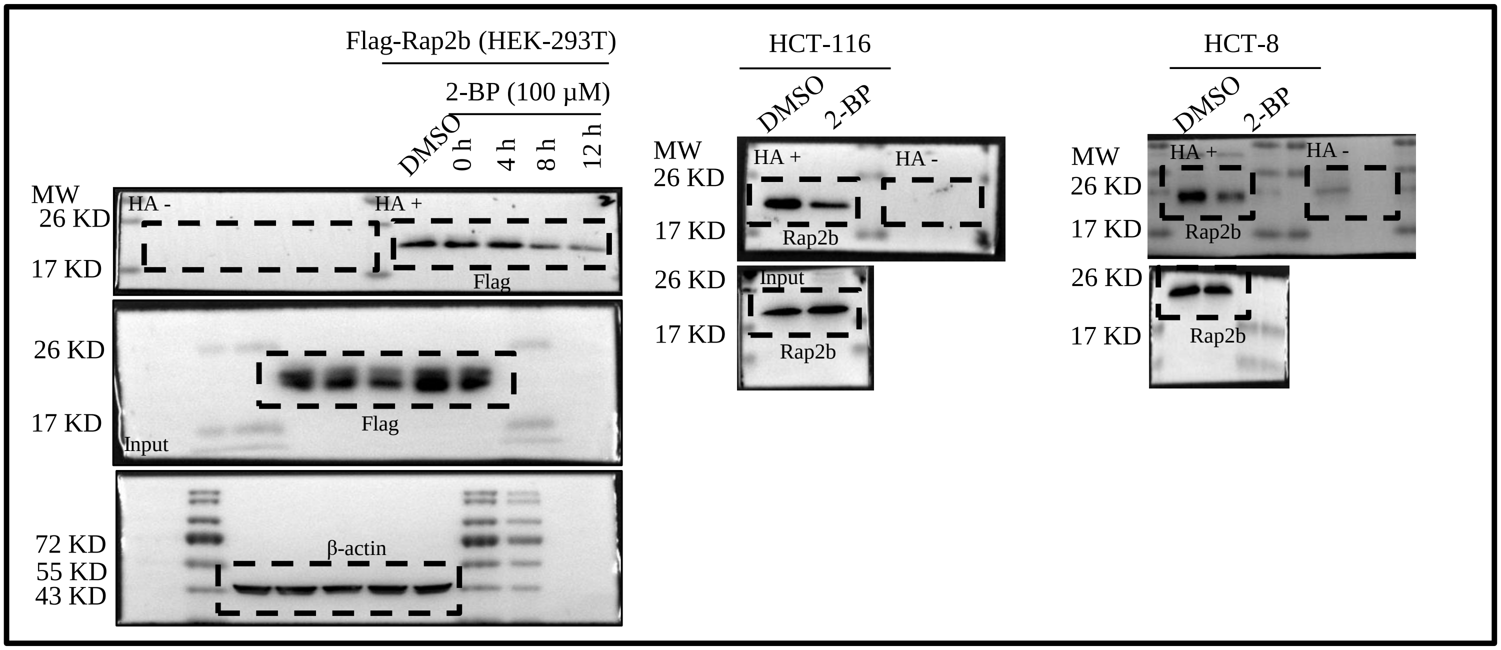

Fig. S11

Uncropped blot of Fig.2B

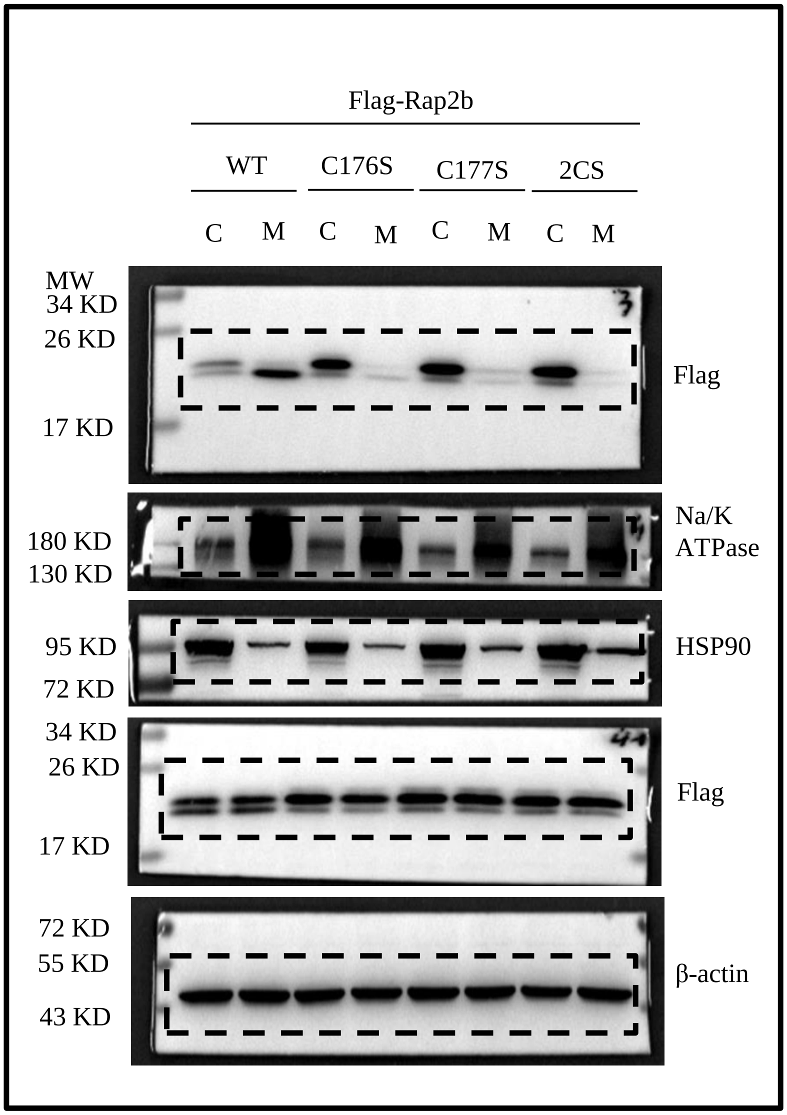

Uncropped blot of Fig.2E

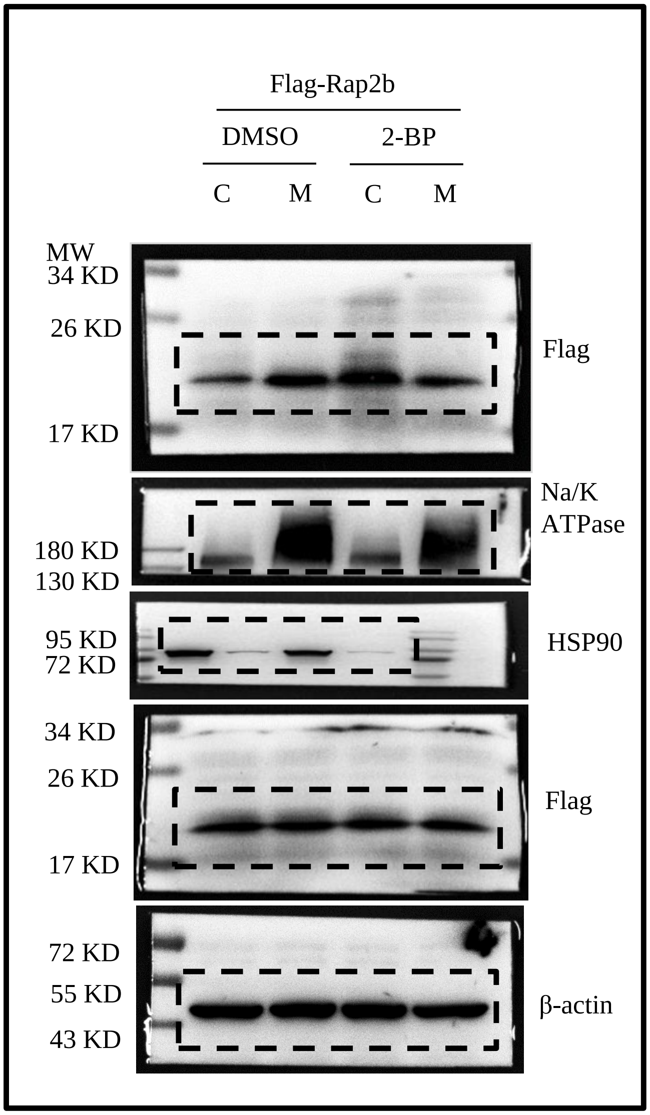

Fig. S12

Uncropped blot of Fig.4A

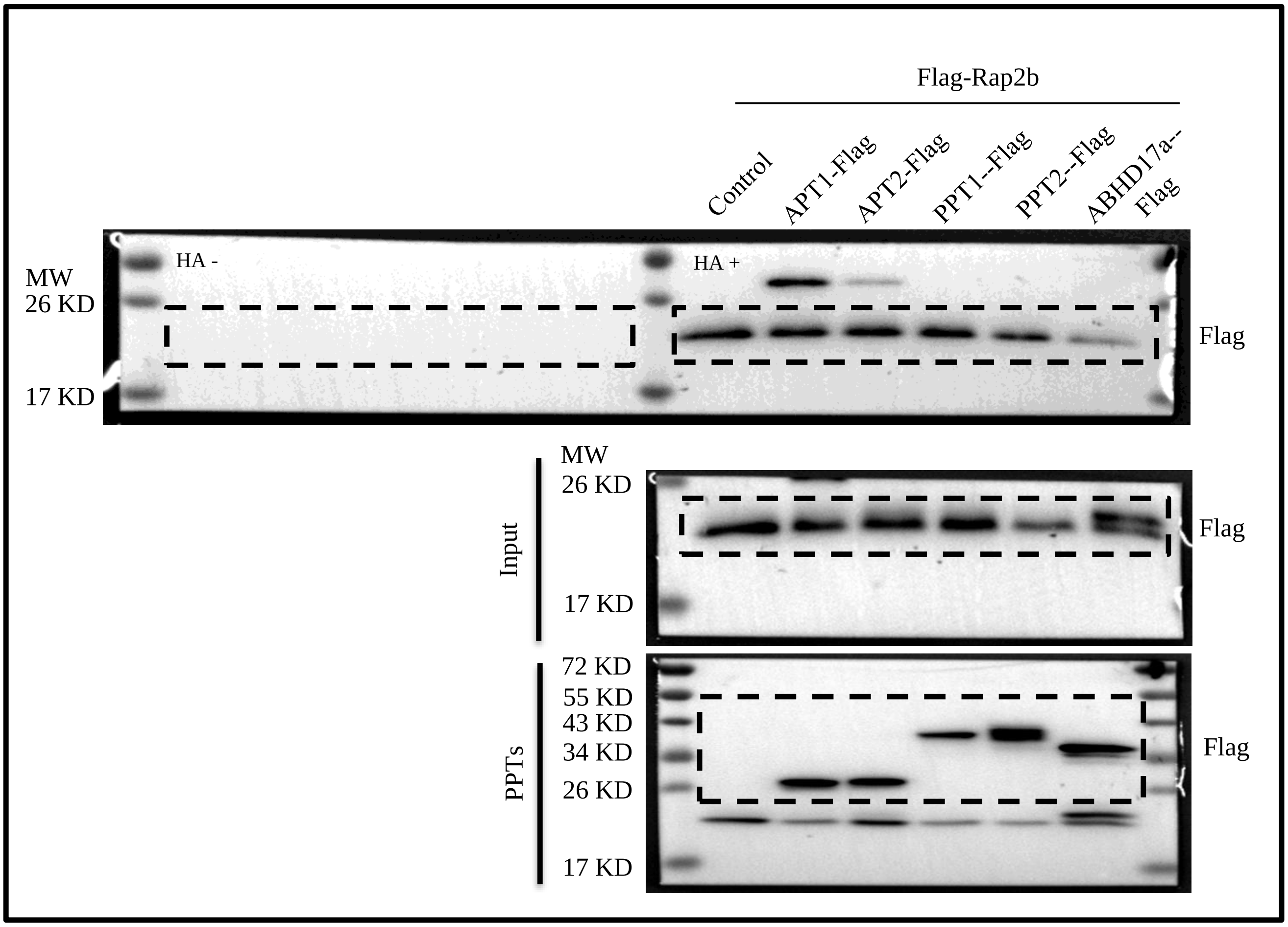

Uncropped blot of Fig.4D

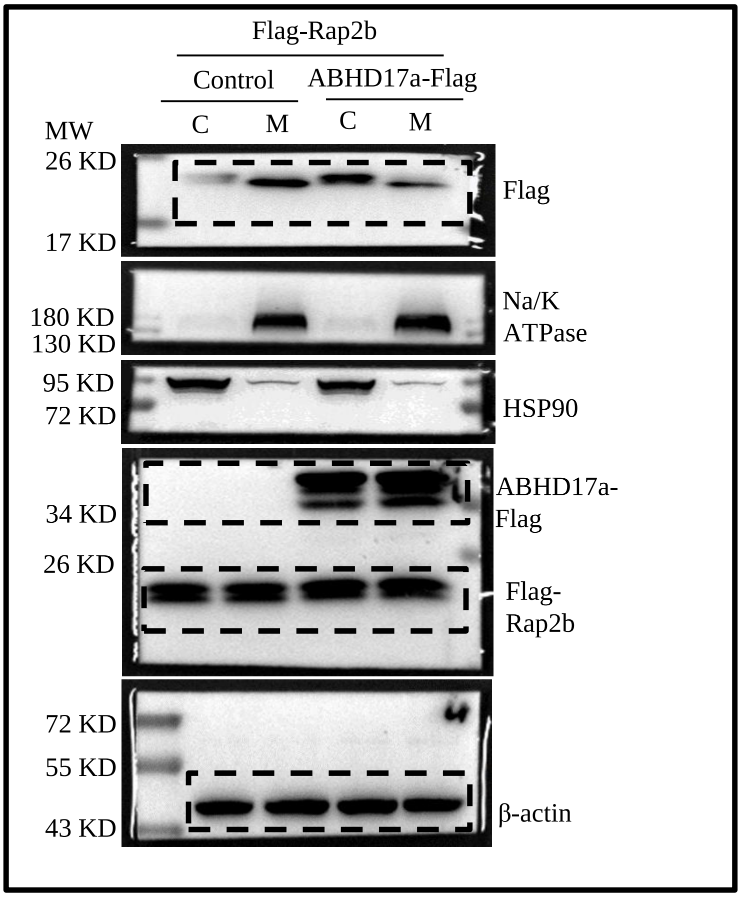

Fig. S13

Uncropped blot of Fig.5A

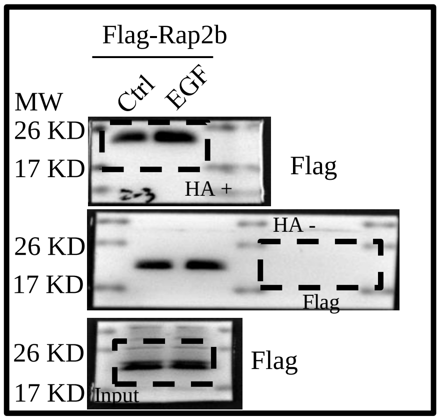

Uncropped blot of Fig.5B

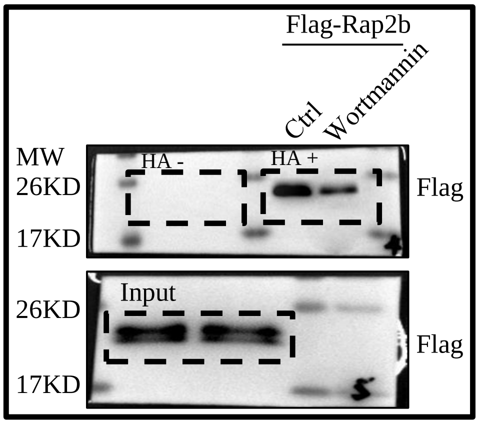

Uncropped blot of Fig.5D

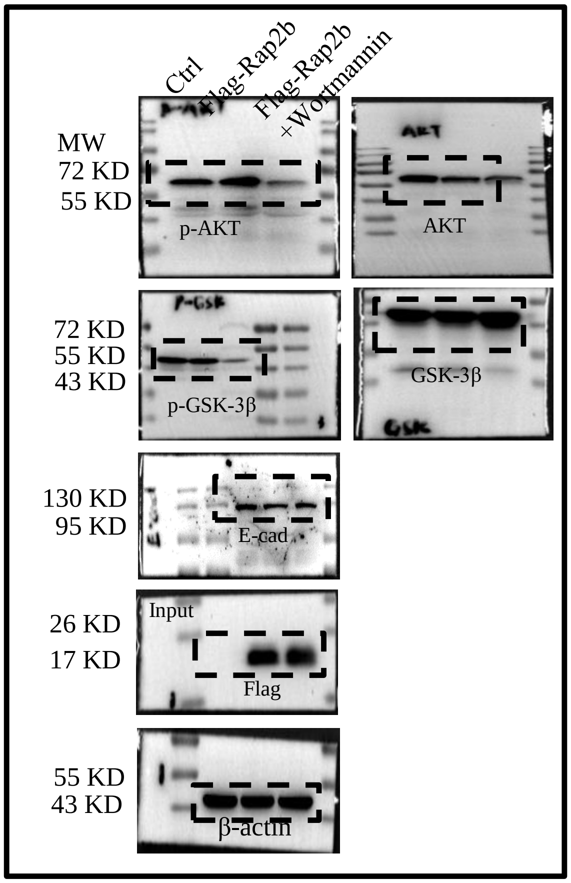

Uncropped blot of Fig.5C

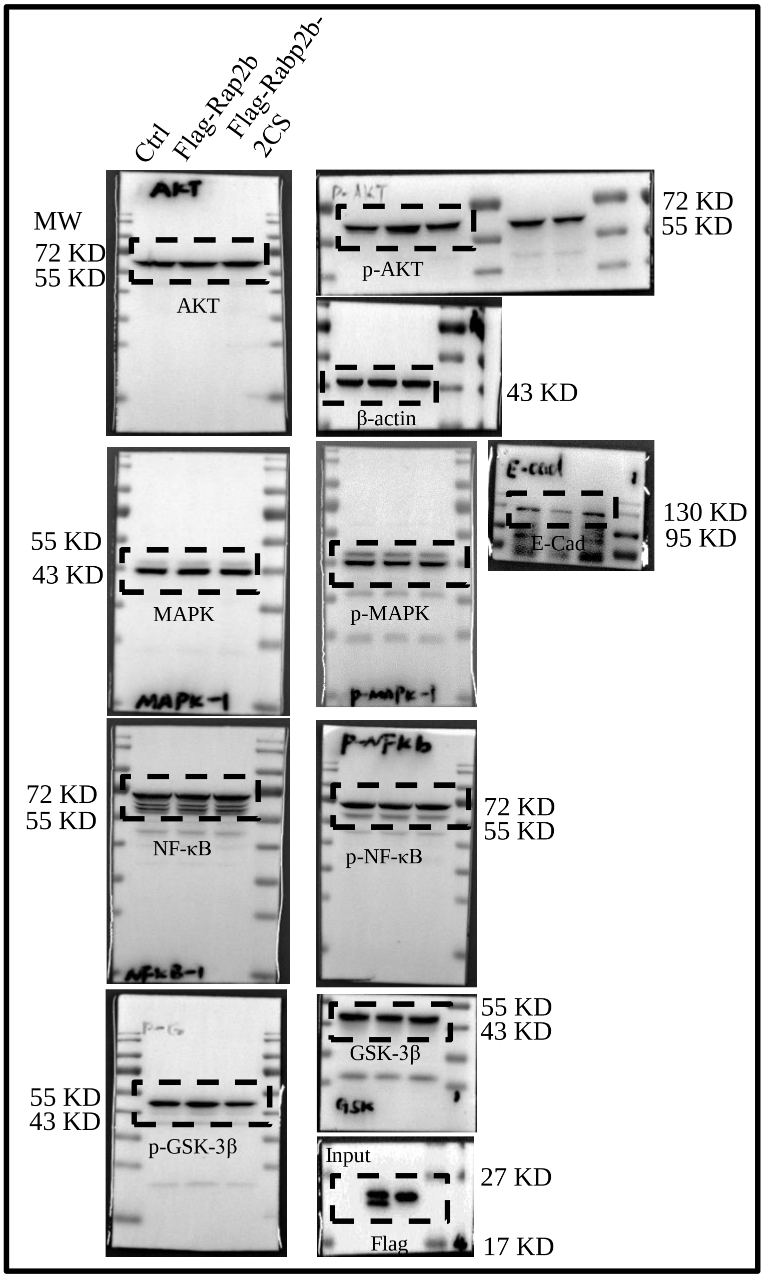

Uncropped blot of Fig.5E

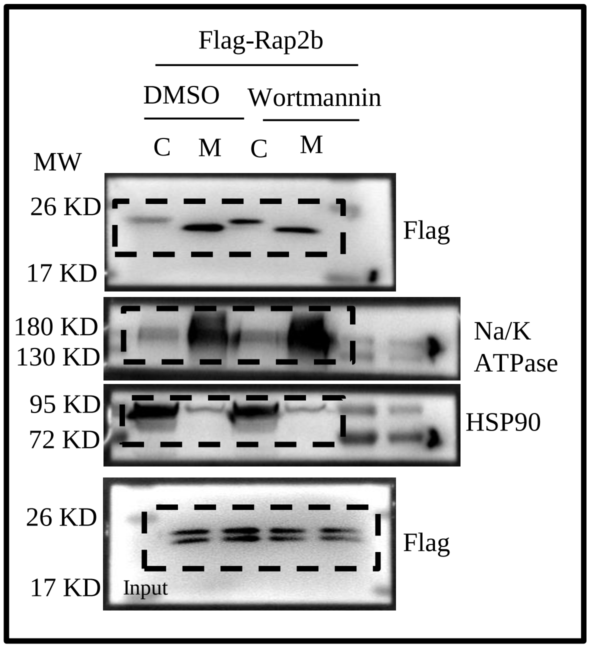

Fig. S14

Uncropped blot of Fig.6A

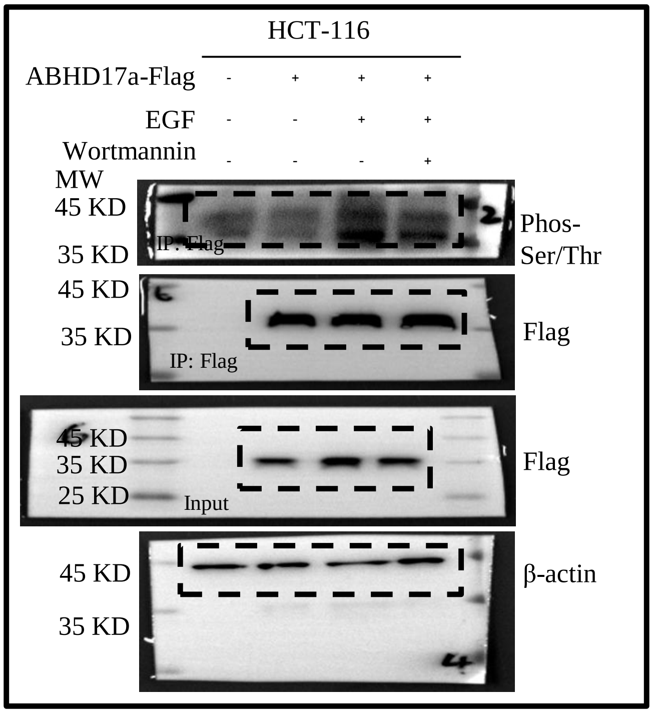

Uncropped blot of Fig.6B

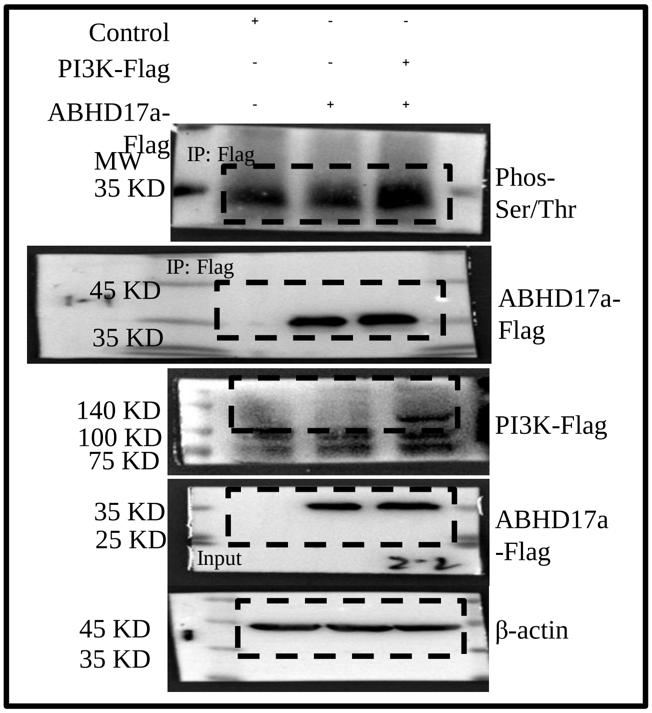

Uncropped blot of Fig.6G

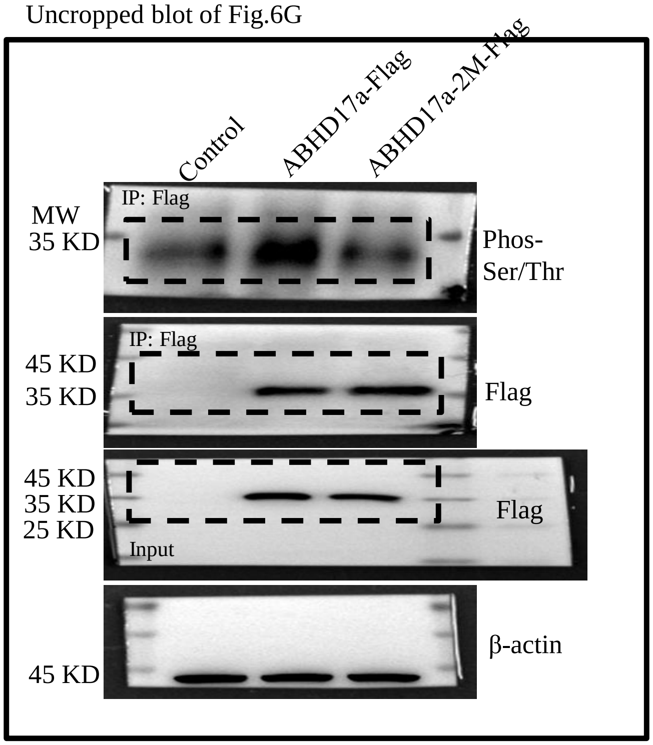

Uncropped blot of Fig.6I

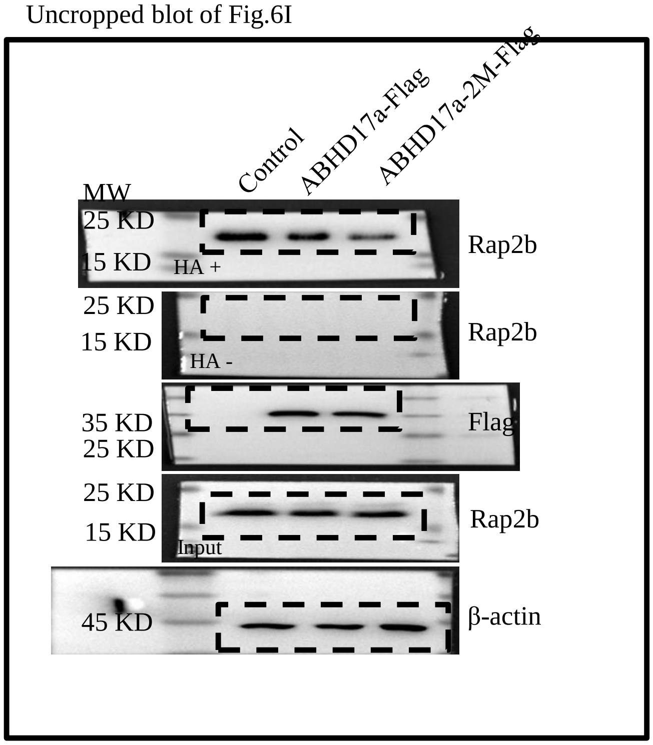

Fig. S15

Uncropped blot of Fig.7A

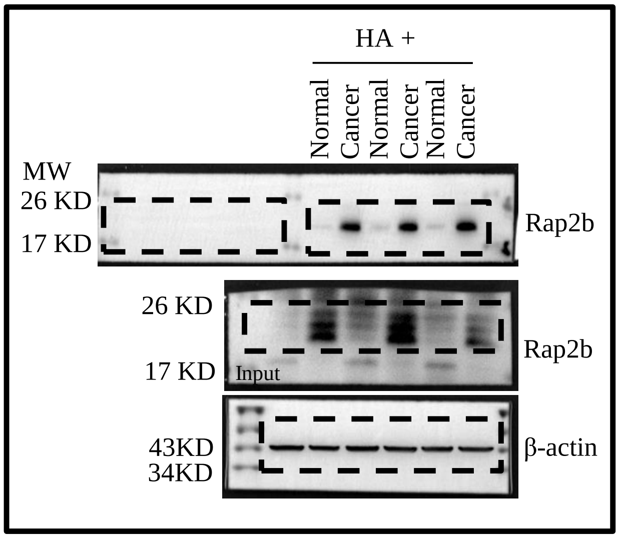

Uncropped blot of Fig.7E

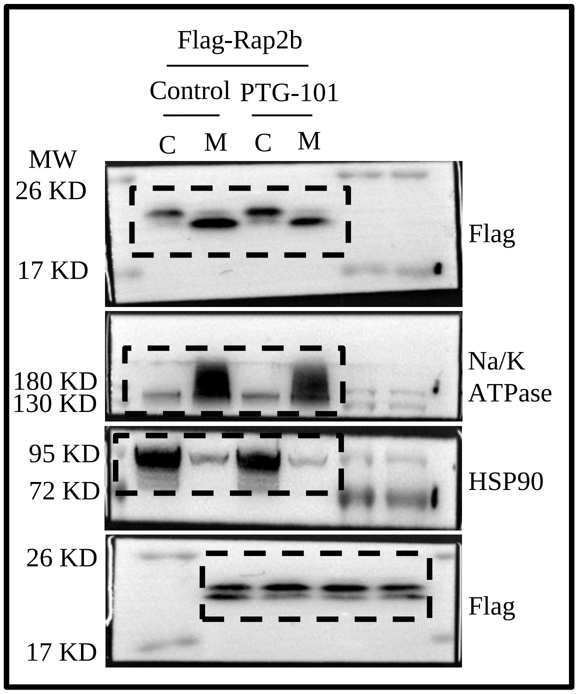

Uncropped blot of Fig.7C

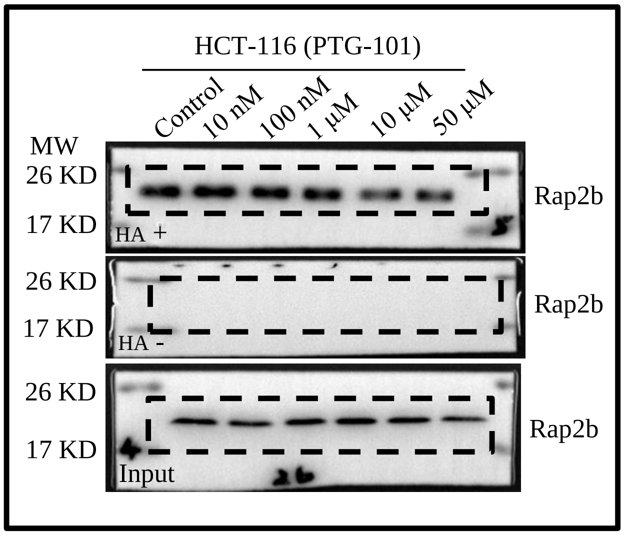

Fig. S16

Uncropped blot of Fig.8F

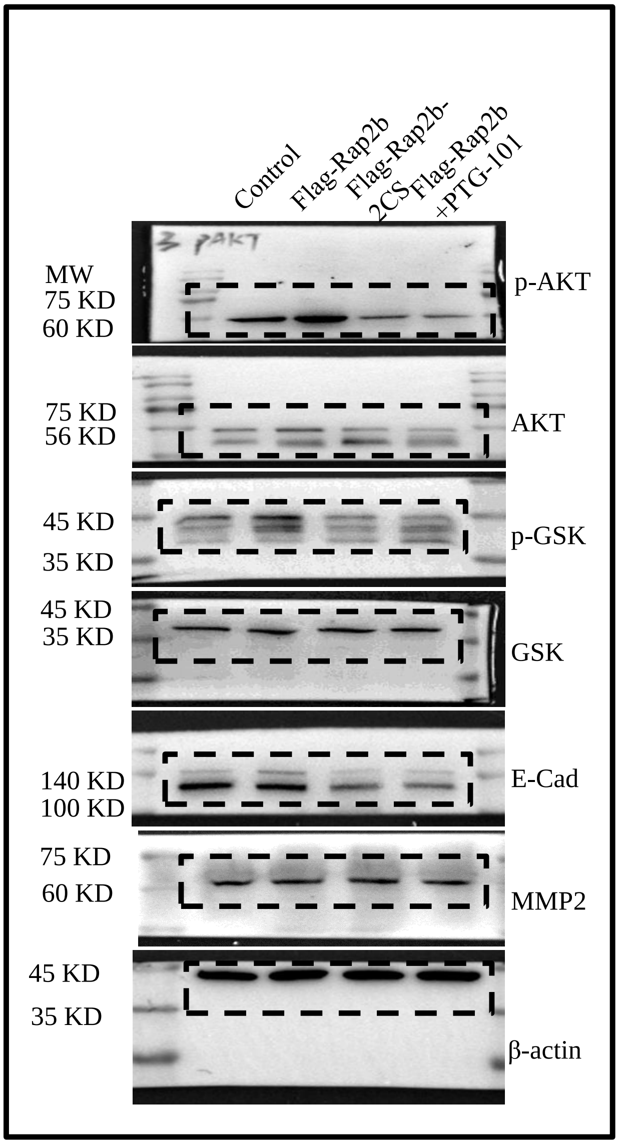

Supplement: Supplementary file 3 — Supplemental figure 1-uncropped western blots [file 41419_2024_7061_MOESM3_ESM.pdf]
