# Supplementary material for: Inhibiting S-palmitoylation arrests metastasis by relocating Rap2b from plasma membrane in colorectal cancer
Source: Cell Death Dis. 2024 Sep 14;15(9):675. doi: 10.1038/s41419-024-07061-2 (PMC11401852; doi:10.1038/s41419-024-07061-2)
Supplement: Supplementary file 4 — Supplemental tables [file 41419_2024_7061_MOESM4_ESM.docx]

Table S1.

The correlation between clinicopathological parameters and Rap2B protein expression in CRC tissues.

| Characteristics | No. of cases | | Rap2b expression | | *P*-value |
| --- | --- | --- | --- | --- | --- |
|  |  |  | Low n (%) | High n (%) |  |
| **Gender** |  | |  |  |  |
| Male | 58 | | 19(32.8%) | 39(67.2%) | 0.896 |
| Female | 59 | | 20(33.9%) | 39(66.1%) |  |
| **Age** |  | |  |  |  |
| <65 | 72 | | 22(30.6%) | 50(69.4%) | 0.420 |
| ≥65 | 45 | | 17(37.8%) | 28(62.2%) |  |
| **T stage** |  | |  |  |  |
| T1-2 | 14 | | 6(42.9%) | 8(57.1%) | 0.420 |
| T3-4 | 103 | | 33(32.0%) | 70(68.0%) |  |
| **Lymph node metastasis** |  | |  |  |  |
| Negative | 47 | | 21(44.7%) | 26(55.3%) | **0.033** |
| Positive | 70 | | 18(25.7%) | 52(74.3%) |  |
| **Distal metastasis** |  | |  |  |  |
| Negative | 92 | | 38(41.3%) | 54(58.7%) | **<0.0001** |
| Positive | 25 | | 1(4.0%) | 24(96.0%) |  |
| **TNM stage** |  | |  |  |  |
| Ⅰ-Ⅱ | 46 | | 21(45.7%) | 25(54.3%) | **0.023** |
| Ⅲ-Ⅳ | 71 | | 18(25.4%) | 53(74.6%) |  |
| **RAS status** |  | |  |  |  |
| Wild-type | 59 | | 21(35.6%) | 38(64.4%) | 0.601 |
| Mutation | 58 | | 18(31.0%) | 40(69.0%) |  |
| **Microsatellite stability status** | |  |  |  |  |
| MSS | 108 | | 36(33.3%) | 72(66.7%) | 1.000 |
| MSI-H | 9 | | 3(33.3%) | 6(66.7%) |  |

Table S2.

Univariate and multivariate analysis of factors associated with DFS in CRC tissues (n = 101)

| Variables | Univariate analysis | | | Multivariate analysis | | |
| --- | --- | --- | --- | --- | --- | --- |
|  | HR | 95%CI | P-value | HR | 95%CI | P-value |
| **Gender** (Male vs. Female) | 0.936 | 0.632-1.386 | 0.741 |  |  |  |
| **Age** (<65 vs. ≥65) | 0.964 | 0.641-1.450 | 0.860 |  |  |  |
| **T stage** (T3-4 vs. T1-2) | 0.859 | 0.486-1.518 | 0.600 |  |  |  |
| **Lymphatic metastasis**  (Positive vs. Negative) | 0.654 | 0.437-0.981 | **0.040** | 0.313 | 0.038-0.592 | 0.281 |
| **Distant metastasis**  (Positive vs. Negative) | 0.185 | 0.098-0.348 | **<0.0001** | 0.252 | 0.128-0.496 | **<0.0001** |
| **TNM stage**  (Ⅲ-Ⅳ vs.Ⅰ-Ⅱ) | 0.629 | 0.419-0.944 | **0.025** | 0.263 | 0.030-2.282 | 0.226 |
| **Rap2b expression**  (High vs. Low) | 0.382 | 0.250-0.582 | **<0.0001** | 0.423 | 0.274-0.653 | **<0.0001** |
